# Prostate Cancer-associated SPOP mutations enhance cancer cell survival and docetaxel resistance by upregulating Caprin1-dependent stress granule assembly

**DOI:** 10.1186/s12943-019-1096-x

**Published:** 2019-11-26

**Authors:** Qing Shi, Yasheng Zhu, Jian Ma, Kun Chang, Dongling Ding, Yang Bai, Kun Gao, Pingzhao Zhang, Ren Mo, Kai Feng, Xiaying Zhao, Liang Zhang, Huiru Sun, Dongyue Jiao, Yingji Chen, Yinghao Sun, Shi-min Zhao, Haojie Huang, Yao Li, Shancheng Ren, Chenji Wang

**Affiliations:** 1 Obstetrics and Gynecology Hospital of Fudan University, State Key Lab of Genetic Engineering, MOE Engineering Research Center of Gene Technology, School of Life Sciences, Key Laboratory of Reproduction Regulation of NPFPC (SIPPR, IRD), Fudan University, Shanghai, 200438 People’s Republic of China; 2 Department of Urology, Shanghai Changhai Hospital, Second Military Medical University, Shanghai, 200433 People’s Republic of China; 3 Department of Urology, Fudan University Shanghai Cancer Center, Fudan University, Shanghai, 200032 People’s Republic of China; 4 Department of Oncology, Shanghai Medical College, Fudan University, Shanghai, 200032 People’s Republic of China; 5 Department of Biochemistry and Molecular Biology, Mayo Clinic College of Medicine, Rochester, MN 55905 USA; 6 Clinical and Translational Research Center, Shanghai First Maternity and Infant Hospital, Tongji University School of Medicine, Shanghai, 200090 People’s Republic of China; 7 Fudan University Shanghai Cancer Center and Institutes of Biomedical Sciences, Shanghai Medical College, Fudan University, Shanghai, 200032 People’s Republic of China; 8 Department of Urology, Inner Mongolia Urological Institute, Inner Mongolia Autonomous Region People’s Hospital, Hohhot, 010017 Inner Mongolia People’s Republic of China

**Keywords:** Prostate cancer, Ubiquitination, Stress granules, Gene mutations

## Abstract

**Background:**

The gene encoding the E3 ubiquitin ligase substrate-binding adaptor SPOP is frequently mutated in primary prostate cancer, but how SPOP mutations contribute to prostate cancer pathogenesis remains poorly understood. Stress granules (SG) assembly is an evolutionarily conserved strategy for survival of cells under stress, and often upregulated in human cancers. We investigated the role of SPOP mutations in aberrant activation of the SG in prostate cancer and explored the relevanve of the mechanism in therapy resistance.

**Methods:**

We identified SG nucleating protein Caprin1 as a SPOP interactor by using the yeast two hybrid methods. A series of functional analyses in cell lines, patient samples, and xenograft models were performed to investigate the biological significance and clinical relevance of SPOP regulation of SG signaling in prostate cancer.

**Results:**

The cytoplasmic form of wild-type (WT) SPOP recognizes and triggers ubiquitin-dependent degradation of Caprin1. Caprin1 abundance is elevated in SPOP-mutant expressing prostate cancer cell lines and patient specimens. SPOP WT suppresses SG assembly, while the prostate cancer-associated mutants enhance SG assembly in a Caprin1-dependent manner. Knockout of SPOP or expression of prostate cancer-associated SPOP mutants conferred resistance to death caused by SG inducers (e.g. docetaxel, sodium arsenite and H_2_O_2_) in prostate cancer cells.

**Conclusions:**

SG assembly is aberrantly elevated in SPOP-mutated prostate cancer. SPOP mutations cause resistance to cellular stress induced by chemtherapeutic drug such as docetaxel in prostate cancer.

## Background

Recurrent mutations in the *SPOP* gene occur in up to 15% of primary prostate cancers [1–4]. Interestingly, the *SPOP*-mutant subtype of prostate cancer has some notable molecular features, including mutual exclusivity with *ERG* rearrangement, elevated levels of DNA methylation, the co-occurrence *CHD1* deletions, and overexpression of *SPINK1* mRNA, supporting the concept that *SPOP*-mutant tumors represent a distinct molecular subclass of prostate cancer [4]. SPOP is one of the adaptor proteins of the CUL3–RBX1 E3 ubiquitin ligase complex. SPOP selectively recruits substrates via its N-terminal MATH domain, while its BTB and BACK domains mediate oligomerization and interaction with CUL3 [5]. SPOP has been linked to the ubiquitination and degradation of multiple substrates, such as SRC-3, AR, DEK, ERG, SENP7, SETD2, NANOG, PD-L1 and BET proteins [6–19]. SPOP also promotes the non-degradative ubiquitination of INF2 and MacroH2A [20, 21]. The vast majority of prostate cancer-associated SPOP mutations identified so far affect evolutionarily conserved residues in the MATH domain, suggesting that these mutations may alter the interaction between SPOP and its substrates [3]. SPOP mutations lead to increased prostate cancer cell proliferation, invasion and immune escape in vivo, implying that SPOP mutations are driving molecular events in prostate cancer initiation and progression [10, 11, 16]. However, limited numbers of SPOP substrates have been identified and functionally explored.

The survival of eukaryotic cells exposed to adverse environmental stress requires a rapid shutoff of global protein synthesis to preserve energy. The stalled messenger ribonucleoprotein particles (mRNPs) are sequestered into non-membrane-enclosed RNA granules called stress granules (SGs) [22]. In addition to stalled mRNPs, SGs also contain ribosomal subunits, eukaryotic translation initiation factors, and RNA-binding proteins such as G3BP1, HuR, Caprin1 and FXR1, which play nucleating roles in SG assembly [23]. SG assembly is an evolutionarily conserved cellular strategy to minimize stress-induced damage. Since the tumor microenvironment is predominantly associated with various forms of stress including hypoxia, low nutrient availability, and high levels of reactive oxygen species, SGs often promote tumorigenesis and progression by supporting cell survival, tumor growth and metastasis [22]. Moreover, several approved chemotherapeutic drugs such as cisplatin, sorafenib and paclitaxel potently stimulate SG assembly, which in turn render cancer cells more resistance to drug treatment [22, 23]. In this regard, inhibition of SG assembly is expected to make cancer cells vulnerable to anti-cancer drugs. Although SGs have been under intense investigation for over a decade, the mechanisms by which genetic alterations lead to enhancement of SG assembly in cancers are poorly understood.

In this study, we demonstrate that SG assembly is elevated in SPOP-mutant prostate cancer cells. Wild-type SPOP, but not the prostate cancer-associated SPOP mutants, suppresses SG assembly by promoting ubiquitination and degradation of Caprin1, a SG nucleating oncoprotein previously found overexpressed in various cancers [24–26]. Increased Caprin1 expression correlates with SPOP mutation status in prostate cancer specimens. Moreover, SPOP mutations enhance cancer cell survival and resistance to docetaxel, a synthetic analog of paclitaxel by upregulating Caprin1-dependent SGs. Our results provide a novel functional link between SPOP mutation and upregulation of SG assembly in prostate cancer.

## Methods

### Cell culture

293 T, LNCaP, 22Rv1, PC-3, and DU-145 cells were obtained from the American Type Culture Collection (ATCC). C4–2 cells were purchased from Uro Corporation. 293 T cells were maintained in DMEM with 10%(v/v) FBS. LNCaP, 22Rv1, PC-3, and DU-145 cells were maintained in RPMI 1640 with 10%(v/v) FBS. For preparation of mouse embryonic fibroblasts (MEF) Cells, Embryos from Pbcre-SPOP^F102C^ homozygous transgenic mice were isolated on E13.5. After the heads, tails, limbs and most of the internal organs were removed, the embryos were cut and typsinized for 20 min, and then seeded into 100 × 20 mm FALCON dishes in 10 mL of complete MEF media. The cells were split in a ratio of 1:2 or 1:3 and then passaged one or two times to obtain a morphologically homogenous culture. With one week of puromycin selection, the MEF cells stably expressed empty vector or SPOP-F102C after being infected with lentiviruses (pTSiN-EV or pTSiN -Cre). Finally, the MEF cells were expanded for further studies. All animal studies were conducted following protocols approved by the Institutional Animal Care and Usage Committee of Mayo Clinic.

### Plasmid constructions

Expression vectors for SPOP has been described previously [7]. The cDNAs of Caprin1 were obtained from Genecopia and subcloned into pCMV-FLAG or Myc vector. SPOP or Caprin1 mutants were generated by KOD-Plus-Mutagenesis Kit (TOYOBO) following the manufacturer’s instructions.

### CRISPR-Cas9 mediated gene knock out stable cell generation

pX459 plasmid was used to clone guide oligos targeting SPOP or Caprin1 gene. C4–2 cells were plated and transfected with pX459 constructs overnight. 24 h after transfection, 1μg/ml puromycin was used to screen cells for 3 days. Living cells were seeded in 96 well plate by limited dilution to isolate monoclonal cell line. The knock out cell clones are screened by Western blot and validated by sanger sequencing. Sequences of gene-specific sgRNAs are listed in Additional file 2: Table S1.

### Tetracycline inducible expression

For generation of the stable cell lines inducibly expressing the FLAG-SPOP, Flp-In T-Rex 293 cells were co-transfected with pOG44 and the SPOP constructs in pcDNA5-FLAG-BirA vectors. 2 days after transfection, cells were selected in hygromycin (100 μg/ml) for 2 weeks, and then the positive clones were pooled and amplified. 1 μg/ml tetracycline was added to stable cells lines for FLAG-SPOP induction.

### Cell cycle and cell death analysis

For cell death analysis, cells were washed 48 h post-treatment with PBS and fixed in 70% ethanol overnight. The cells were washed again with PBS, stained with propidium iodide and analyzed by flow cytometry. For cell cycle analysis, cells were harvested and washed, followed by propidium iodide staining (10 μg/ml) with 0.3% triton permeation and RNase treatment. Results are representatives of three independent experiments with triplicate samples for each condition.

### Real-time reverse transcription PCR (qRT-PCR)

Total RNA was isolated from cells using the TRIzol reagent (Tiangen), and cDNA was reversed-transcribed using the Superscript RT kit (TOYOBO) following the manufacturer’s instructions. PCR amplification was performed using the SYBR Green PCR master mix Kit (TOYOBO). All quantitations were normalized to the level of endogenous control *GAPDH*. The sequences of Primers for qRT-PCR were listed in Additional file 2: Table S1.

### Ribopuromycylation assay

Ribopuromycylation assay was described previously [27]. In brief, cells were unstressed or stressed as indicated. 5 min before fixation, Puromycin and EM were added to a final concentration of 9 and 91 μM, respectively, and the incubation continued for 5 min. Cells were then lysed subjected to Western blotting using anti-puromycin antibody. Cells with DMSO treatment were used as negative control.

### Nuclear/cytoplasmic fractionation

5 × 10^6^ C4–2 cells were collected, washed twice with cold PBS and lysed in cold hypotonic buffer (10 mM HEPES pH 7.9, 10 mM KCl, 0.1 mM EDTA, 0.1 mM EGTA, 1 mM DTT) supplemented with complete protease inhibitor cocktail without EDTA (Roche) on ice for 15 min. NP-40 was added to a final concentration of 0.625% and cells were vortexed vigorously for 10 s. Samples were centrifuged for 30 s at 16000 g and the supernatants were harvested as cytoplasmic fraction. The nuclear pellets were then resuspended in cold hypertonic buffer (20 mM HEPES pH 7.9, 0.4 M NaCl, 1 mM EDTA, 1 mM EGTA, 1 mM DTT) supplemented with complete protease inhibitor cocktail (Roche). The samples were incubated at 4 °C for 15 min with agitation. The supernatants were collected after centrifugation for 5 min at 16000 g as nuclear proteins. Cytoplasmic and nuclear proteins were frozen in 5x sample buffer at − 20 °C until use.

### Polysome profile analysis

Cells were washed with cold HBSS, scrape-harvested directly into lysis buffer (10 mM Hepes, pH 7.5, 125 mM KCl, 5 mM MgCl2, 1 mM DTT, 100 μg/ml cycloheximide, 100 μg/ml heparin, and 1% NP-40), and supplemented with RNAsin Plus inhibitor (Promega) and HALT phosphatase and protease inhibitors (Thermo). Lysates were rotated at 4 °C for 15 min, cleared by centrifugation for 10 min at 12,000 g, and supernatants were loaded on preformed 17.5–50% sucrose gradients made in gradient buffer (10 mM Hepes, pH 7.5, 125 mM KCl, 5 mM MgCl_2_, and 1 mM DTT). Samples were centrifuged in a Beckman SW140 Ti rotor for 2.5 h at 35,000 rpm and then eluted using a Brandel bottom-piercing apparatus connected to an ISCO UV monitor, which measured the eluate at OD 254 nm.

### EdU incorporation assay

The effects of Caprin1 expression on cell proliferation were determined by EdU incorporation assay using Cell-Light™ EdU Apollo®567 In Vitro Imaging Kit (Ribobio). Briefly, the EdU was added to each well with a finial concentration of 50 μM. After 2 h, cells were fixed with 4% paraformaldehyde in PBS. Fixed 30 min at room temperature, cells were incubated with 2 mg/ml glycine for 5 min. After washed in PBST (PBS containing 0.1% Triton X-100) for 3 times, cells were incubated with 1 X Apollo solution for 30 min at room temperature in the dark. Finally, cells were subjected to nuclear staining with DAPI for 30 min and then observed using Olympus fluorescence microscopy.

### Cell proliferation assay

Cell proliferation rate was determined using Cell Counting Kit-8 (CCK-8) according to the manufacturer’s protocol (Dojindo). Briefly, the cells were seeded onto 96-well plates at a density of 1000 cells per well. During a 2 to 8-day culture periods, 10 μl of the CCK-8 solution was added to cell culture, and incubated for 2 h. The resulting color was assayed at OD 450 nm using a microplate absorbance reader (Bio-Rad). Each assay was carried out in triplicate.

### Migration assay

Cell migration was determined by Transwell (Costar) migration assay. C4–2 cells were precultured in serum-free medium for 48 h. For migration assay, 3 × 10^4^ cells were seeded in serum-free medium in the upper chamber, and the lower chamber was filled with RPMI1640 containing 5% FBS. After 48 h, the non-migrating cells on the upper chambers were carefully removed with a cotton swab, and migrated cells underside of the filter stained and counted in nine different fields.

### Immunofluorescence and confocal microscopy

For immunofluorescence, cells were plated on chamber slides, fixed with 4% paraformaldehyde at room temperature for 30 min. After washing with PBS, cells were permeabilized with 0.1% Triton X-100 in PBS for 15 min at room temperature. Cells were then washed with PBST, blocked with 5% donkey serums in PBS for 1 h, and incubated with primary antibodies in PBS at 4 °C for overnight in the dark. After washing with PBST, fluorescence-labelled secondary antibodies were applied and DAPI was counterstained for 1 h at room temperature in the dark. Slides were mounted in ProlongGold (Invitrogen). Cells were visualized and imaged using a confocal microscope (LSM710, Zeiss) with a 63*/1.4NA Oil PSF Objective. The Stress granules index was quantified using Image J by computing the total stress granules area in relation to the total cell area in a field with at least 10 cells. The analysis results were carried out in triplicate from three different fields.

### SPOP mutation detection, immunohistochemistry (IHC) and RT-qPCR

Treatment-naive prostate cancer and matched benign tissues were collected from the radical prostatectomy series at Shanghai Changhai Hospital, and the institutional review board of the hospital approved the experimental protocols. Haematoxylin and eosin (H&E) slides of frozen and formalin-fixed paraffin-embedded (FFPE) human tumor tissues and matched benign tissues were examined by a general pathologists and a genitourinary pathologist to confirm histological diagnosis, Gleason score and verify the high-density cancer foci (> 80%) of the selected tumor tissue. The frozen blocks for DNA/RNA extraction were examined by the pathologists as described above, followed by consecutive ten 10-μm sections of each tumor. These qualified samples were then used for DNA/RNA isolation. FFPE tissues were used for immunohistochemistry (IHC). The methods of SPOP mutation detection by sanger sequencing and IHC analysis, RNA extraction from FFPE patient tissues and RT-qPCR analysis were described previously [17].

### Generation of prostate cancer xenografts in mice

All experimental protocols were approved in advance by the Ethics Review Committee for Animal Experimentation of Fudan University. 4–6 week old BALB/c nu/nu mice obtained from SLAC Laboratory Animal Co., Ltd. were bred and maintained in our institutional pathogen-free mouse facilities. 5 × 10^6^ indicated C4–2 prostate cancer cells were suspended in 100 μl of PBS buffer and injected into the flanks of male nude mice (four mice for each group). At the end of 3 weeks, mice were killed and in vivo solid tumors were dissected and weighed.

### Statistical analysis

The statistical calculations were performed using GraphPad Prism software. All data are shown as mean values ± SD for experiments performed with at least three replicates. The difference between 2 groups was analyzed using paired Student’s t-test unless otherwise specified. * represents *p* < 0.05; ** represents *p* < 0.01; *** represents *p* < 0.001.

## Results

### Identification of Caprin1 as a novel SPOP interactor

By performing a yeast two-hybrid screen in a human fetal brain cDNA library using full length SPOP as bait, we identified 32 clones corresponded to Caprin1 protein. Caprin1 is a core component of SG. We explored whether Caprin1 is an authentic SPOP substrate, and whether its physiological function is dysregulated in SPOP-mutated prostate cancer.

**Fig. 1:**
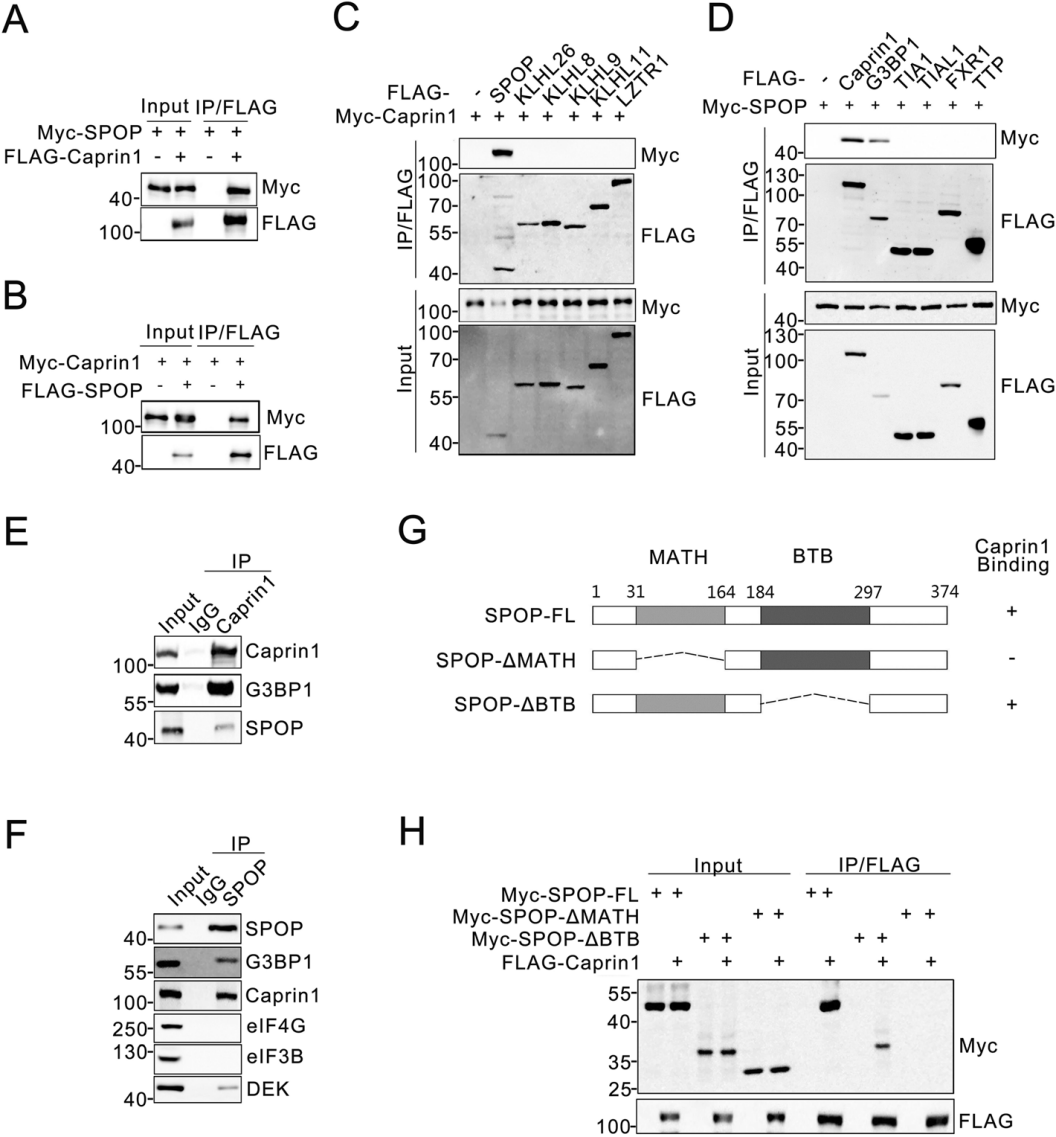
SPOP interacts with Caprin1. **a**-**d** Western blot of indicated proteins in WCLs and co-IP samples of anti-FLAG antibody obtained from 293 T cells transfected with indicated plasmids. **e** Western blot of indicated proteins in WCLs and co-IP samples of IgG or anti-Caprin1 antibody obtained from the cell extracts of C4–2 cells treated with 20 μM of MG132 for 8 h. **f** Western blot of indicated proteins in WCL and co-IP samples of IgG or anti-SPOP antibody obtained from the cell extracts of C4–2 cells treated with 20 μM of MG132 for 8 h. **g** Schematic representation of SPOP deletion mutants. Binding capacity of SPOP to Caprin1 is indicated with the symbol. **h** Western blot of indicated proteins in WCLs and co-IP samples of anti-FLAG antibody obtained from 293 T cells transfected with indicated plasmids

We first examined whether SPOP could interact with Caprin1 in cells. We coexpressed FLAG–Caprin1 and Myc–SPOP constructs in 293 T cells and performed co-immunoprecipitation (co-IP) analysis with an anti-FLAG antibody. As shown in Fig. 1a, Myc-SPOP was successfully co-immunoprecipitated by FLAG-Caprin1, suggesting an interaction between the two exogenously expressed proteins. This result was confirmed by reciprocal co-IP assay (Fig. 1b). Only SPOP, but not the other CUL3-based BTB-domain-containing adaptors we examined (KLHL26, KLHL8, KLHL9, KLHL11 and LZTR1), specifically interacted with Caprin1 (Fig. 1c). We noted that G3BP1, but not other SG nucleating proteins (TIA1, TIAL1, FXR1 and TTP), interacted with SPOP (Fig. 1d). Next, we investigated the potential binding between endogenous SPOP and Caprin1. We performed co-IP using an anti-Caprin1 antibody in C4–2 cell lysate. Caprin1 was able to immunoprecipiate SPOP and G3BP1, a known interactor of Caprin1 (Fig. 1e). Reciprocally, SPOP was able to immunoprecipiate Caprin1 and G3BP1, but not the SG components eIF4G1 or eIF3B (Fig. 1f). These results suggest that SPOP can interact with the Caprin1 and G3BP1 proteins at the endogenous level.

SPOP contains two structural domains, one is the substrate-binding MATH domain at the N-terminus and the other is the CUL3-binding BTB domain at the C-terminus. To determine which domain of SPOP mediates its interaction with Caprin1, we generated SPOP-ΔBTB and ΔMATH mutants by deleting these two mutants individually (Fig. 1g). A co-IP assay was performed to determine the ability of ectopically expressed Caprin1 to bind these mutants. While full-length SPOP (SPOP-FL) and SPOP-ΔBTB efficiently interacted with Caprin1, the interaction was abolished by SPOP-ΔMATH (Fig. 1h). Thus, SPOP interacts with Caprin1 through the MATH domain.

### SPOP promotes Caprin1 degradation and ubiquitination

**Fig. 2:**
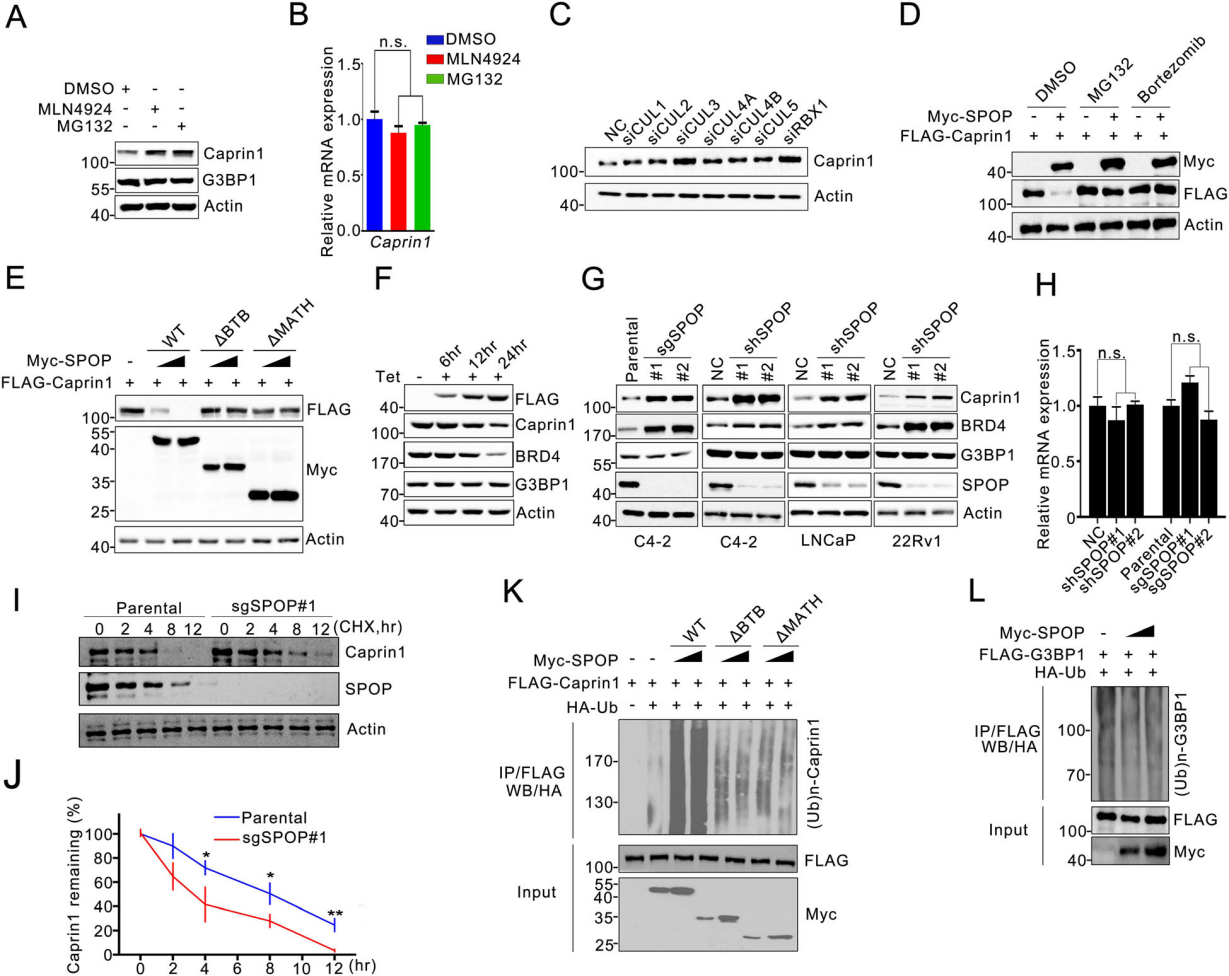
SPOP promotes Caprin1 degradation and ubiquitination. **a** Western blots of indicated proteins in WCLs from C4–2 cells treated with DMSO, MG132 (20 μM) or with MLN4924 (100 nM) for 8 h. **b** RT-qPCR assessment of Caprin1 mRNA expression in C4–2 cells treated with DMSO, MG132 (20 μM) or with MLN4924 (100 nM) for 8 h. The mRNA level of GAPDH was used for normalization. Data are shown as means ± SD (*n* = 3). **c** Western blot of indicated proteins in WCLs from C4–2 cells transfected with indicated siRNAs. **d** Western blot of indicated proteins in WCLs from 293 T cells transfected with indicated plasmids with DMSO, MG132 (20 μM) or with Bortezmib (20 nM) for 8 h. **e** Western blot of indicated proteins in WCLs from 293 T cells transfected with indicated plasmids. **f** Western blot of indicated proteins in WCLs from FLAG-SPOP inducible Flp-In T-Rex 293 cells treated with tetracycline with indicated times. **g** Western blot of indicated proteins in WCLs from C4–2 cells with SPOP knockout through CRISPR/Cas9 methods. Parental C4–2 cells were used as the control; Western blot of indicated proteins in WCLs from C4–2, LNCaP and 22Rv1 cells infected with lentivirus expressing SPOP-specific shRNA or negative control (NC). **h** RT-qPCR measurement of Caprin1 mRNA expression in C4–2 cells infected with lentivirus expressing SPOP-specific shRNA or NC; RT-qPCR measurement of Caprin1 mRNA expression in parental and SPOP-KO C4–2 cells. Data are shown as means ± SD (*n* = 3). **i**, **j** Western blot of indicated proteins in WCLs of C4–2 cells infected with lentivirus expressing SPOP-specific shRNA or NC for 48 h and then treated with 50 μg/ml cycloheximide (CHX) and harvested at different time points (**i**). At each time point, the intensity of Caprin1 was normalized to the intensity of actin and then to the value at 0 h (**j**). Similar results were obtained from two independent experiments. **k**, **l** Western blot of the products of in vivo ubiquitination assays from 293 T cells transfected with the indicated plasmids and treated with 20 μM MG132 for 8 h

The Caprin1 protein plays a critical role in SG assembly, but little is known about its functional regulation by post-translational modifications. Caprin1 was ubiquitnously expressed in various prostate cancer cell lines (Additional file 1: Figure S1). Treatment of C4–2 cells with the proteasome inhibitor MG132 inevitably increased Caprin1 protein levels but not the corresponding mRNA level (Fig. 2a, b). MLN4924, a small-molecule inhibitor of NEDD8-activating enzyme that is required for activation of CRL complexes, also caused accumulation of Caprin1 protein, but not mRNA (Fig. 2a, b). We depleted RBX1 or each Cullin adaptor, including CUL1–5 in C4–2 cells by small interfering RNA (siRNA) and found that only RBX1 or CUL3 depletion led to a marked increase in the abundance of Caprin1 protein, suggesting that Caprin1 protein stability is subjected to be regulated by a CUL3–RBX1 ubiquitin ligase complex (Fig. 2c). We found that expression of SPOP markedly decreased Caprin1 protein levels, and this effect was completely reversed by treatment with proteasome inhibitors MG132 or Bortezomib (Fig. 2d). Moreover, only WT SPOP, but not substrate-binding- and CUL3-binding-deficient mutants (∆MATH and ∆BTB), degraded Caprin1 protein (Fig. 2e). To characterize the effect of SPOP on endogenous Caprin1, Tet-on-inducible Flp-In 293 T-REx stable cell lines conditionally expressing FLAG-SPOP were generated. Induction of FLAG-SPOP by tetracycline treatment led to decreased expression of endogenous Caprin1 as well as BRD4, a known SPOP substrate in a time-dependent manner (Fig. 2f). Depletion of SPOP by short hairpin RNA (shRNA)-mediated knockdown or CRISPR/Cas9-mediated knockout in multiple prostate cancer cell lines resulted in a marked increase in the steady-state levels of endogenous Caprin1 (Fig. 2g, Additional file 1: Figure S2A-D).). Notably, Caprin1 mRNA levels in SPOP knockown or knockout cells were similar to those in control cells (Fig. 2h). Knockout of SPOP remarkably prolonged the half-life of Caprin1 protein in C4–2 cells (Fig. 2i, j). Expression of WT SPOP, but not the ∆MATH or ∆BTB mutant, induced robust polyubiquitination of Caprin1 (Fig. 2k).

Although G3BP1 interacted with SPOP, depletion or overexpression of SPOP did not alter the G3BP1 protein level (Fig. 2f, g). Moreover, SPOP overexpression had no impact on G3BP1 ubiquitination (Fig. 2l). These results suggest that G3BP1 is not a ubiquitination substrate of SPOP. Taken together, our data demonstrate that the SPOP–CUL3–RBX1 E3 ubiquitin ligase complex regulates Caprin1 protein stability through ubiquitin-dependent proteasomal degradation in prostate cancer cells.

### The SPOP-binding consensus motif in Caprin1 is required for SPOP-mediated Caprin1 degradation and ubiquitination

Previous studies have reported that one or several SPOP-binding consensus SBC motifs are present in SPOP substrates [5]. We sought to determine the protein sequence of Caprin1 responsible for SPOP-binding. To this end, we first deduced the minimal interacting region from the four SPOP-bound fragments of Caprin1 obtained from the yeast two-hybrid screen. We found Caprin1 (amino acids 380~512) corresponds to the smallest region necessary for SPOP interaction (Additional file 1: Fig. S3A). Next, we performed a protein motif search in this region and discovered a perfectly matched SBC motif. Caprin1 gene is present only in vertebrates. The SBC motif is conserved in human, mouse and chicken, but not in frog and fish (Additional file 1: Figure S3B). Interestingly, this motif is very similar to the SBC motif present in several known SPOP substrates (Additional file 1: Figure S3B). To examine whether this potential motif is required for SPOP–Caprin1 interaction, we generated an Caprin1 mutant in which the motif sequence was deleted. 293 T cells were co-transfected with SPOP and WT Caprin1 or ΔSBC mutant. A co-IP assay demonstrated that SPOP only bound to the WT Caprin1, but not the ΔSBC mutant although they were expressed at comparable levels (Additional file 1: Figure S3C), suggesting that the SBC motif of Caprin1 is required for SPOP binding. Moreover, deletion of the SBC motif in the Caprin1 protein not only abolished SPOP-mediated degradation and ubiquitination, but also substantially prolonged the half-life of the mutant in 293 T cells (Additional file 1: Figure S3D-G). Collectively, we identify a conserved SBC motif present in Caprin1 that is indispensable for SPOP-dependent ubiquitination and degradation.

### Prostate cancer-associated SPOP mutants are defective in promoting Caprin1 ubiquitination

**Fig. 3:**
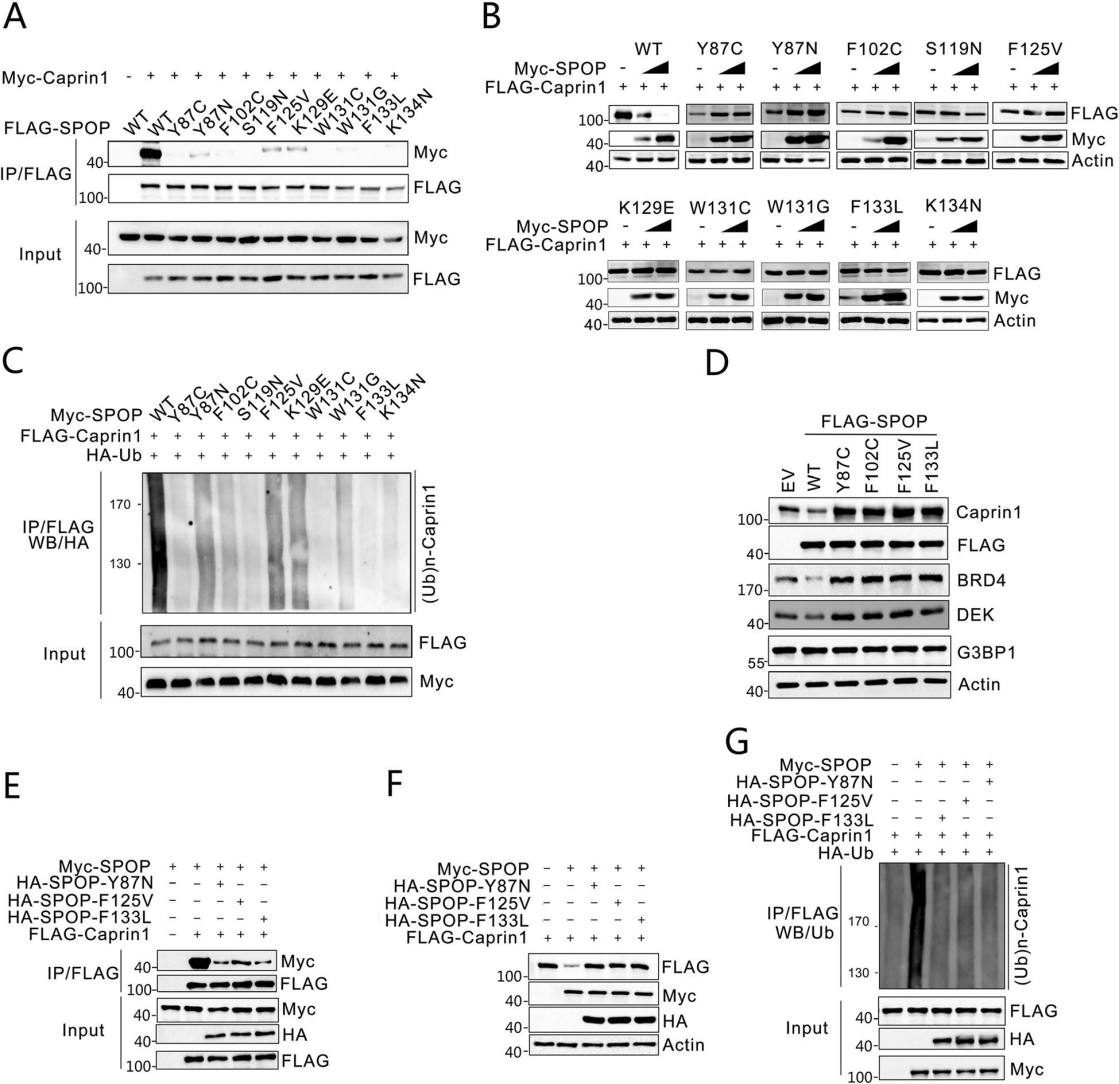
Prostate cancer-associated mutants of SPOP are defective in promoting Caprin1 degradation and ubiquitination. **a** Western blot of indicated proteins in WCLs and samples from co-IP with anti-FLAG antibody in 293 T cells transfected with the indicated plasmids and treated with 20 μM MG132 for 8 h. **b** Western blot of indicated proteins in WCLs from 293 T cells transfected with the indicated plasmids. **c** Western blot of the products of in vivo ubiquitination assays from 293 T cells transfected with the indicated plasmids and treated with 20 μM MG132 for 8 h. **d** Western blot of the indicated proteins in WCLs from C4–2 cells infected with empty vector (EV) or lentivirus expressing wild-type or mutant SPOP. **e** Western blot of indicated proteins in WCLs and co-IP samples of anti-FLAG antibody obtained from 293 T cells transfected with indicated plasmids and treated with 20 μM MG132 for 8 h. **f** Western blot of indicated proteins in WCLs from 293 T cells transfected with the indicated plasmids. **g** Western blot of the products of in vivo ubiquitination assays from 293 T cells transfected with the indicated plasmids and treated with 20 μM MG132 for 8 h

The vast majority of the SPOP mutations detected thus far in prostate cancer primarily occur in the MATH domain, which is responsible for substrate binding [3]. According to a study about SPOP mutation frequency in prostate cancer across demographically diverse patient cohorts, the most frequently mutated residue was F133 (50%) followed by Y87 (15%), W131 and F102 (9%), F125 (3%), and K129, F104, and K135 (2%), other mutated residues are less frequent [27]. We postulated that prostate cancer-associated mutants of SPOP may be defective in mediating Caprin1 destruction. We examined the interactions between cancer-associated mutants of SPOP and Caprin1 by co-IP assays. As shown in Fig. 3a, the Caprin1 binding ability of all SPOP mutants was severely impaired compared with wildtype SPOP. SPOP-mediated degradation and ubiquitination of Caprin1 protein were also markedly attenuated for these mutants (Fig. 3b, c). Stable expression of a few hotspot mutants of SPOP in C4–2 Cells failed to degrade Caprin1 protein and instead led to elevated endogenous levels of Caprin1, showing a dominant-negative effect similar to that on the SPOP known substrates such as DEK and BRD4 (Fig. 3d). Indeed, we found that coexpression of SPOP mutants (Y87N, F125 V or F133 L) reduced the interaction between WT SPOP and Caprin1 (Fig. 3e), and suppressed WT SPOP-induced Caprin1 degradation and ubiquitination **(**Fig. 3f, g). These data indicate that prostate cancer-associated SPOP mutations result in the stabilization of Caprin1 protein in prostate cancer cells.

### Cytoplasmic but not nuclear SPOP promotes Caprin1 degradation and ubiquitination

Caprin1 is a cytoplasmic RNA-binding protein. Our previous studies showed that SPOP was either localized exclusively in the nucleus as speckles, or in both the cytoplasm and nucleus, indicating that SPOP constantly shuttles between the cytoplasm and nucleus in cells. We investigated the subcellular localization at which SPOP–Caprin1 interaction occurs. Myc-Caprin1 was diffusely localized in the cytoplasm (Additional file 1: Figure S4A). When Myc-Caprin1 and HA-SPOP were coexpressed in cells, HA–SPOP was localized in both the cytoplasm and the nucleus in approximately 30% of cells whereas Myc–Caprin1 was primarily present as speckles in the cytoplasm and colocalized with SPOP (Additional file 1: Figure S4A). Notably, SPOP/Caprin1 was not colocalized with eIF4G,a well-established marker of SG, suggesting that these speckles are not stress granules. In the remaining cells, HA-SPOP was localized exclusively in the nucleus, and SPOP was not co-localized with cytoplasmic Caprin1 (Additional file 1: Figure S4A). We found that SPOP lacking the NLS sequence (SPOP-ΔNLS) accumulated exclusively in the cytoplasm as a punctate pattern and perfectly colocalized with Myc-Caprin1. Moreover, deletion of the SBC motif (ΔSBC) in Caprin1 did not alter its diffusely cytoplasmic localization, but the SPOP-WT or ΔNLS mutant-induced speckle pattern of Caprin1 was not observed (Additional file 1: Figure S4A). Next, we investigated the impact of prostate cancer-associated mutants of SPOP on Caprin1 localization. To this end, we focused on two hotspot mutations, F125 V and 133 L. Our previous studies showed that the cytoplasmic retention ability of these SPOP mutants is impaired and they are exclusively localized as nuclear speckles in nearly 100% of cells [20]. Accordingly, we found that overexpression of these two SPOP mutants had no impact on the cytoplasmic localization of Myc-caprin1 (Additional file 1: Figure S4A). Moreover, two prostate cancer-associated SPOP mutants lacking the NLS sequence (SPOP-ΔNLS- F125 V and SPOP-ΔNLS- F133 L) accumulated exclusively in the cytoplasm as a punctate pattern similar to SPOP-ΔNLS, but these mutants did not co-localize with Myc–Caprin1, possibly because of an impaired interaction with Caprin1 (Additional file 1: Figure S4A). Furthmore, the subcellular localzaiton of endogenous or transfected SPOP/Caprin1 (WT or mutants) were validated by the nuclear/cytoplasmic fractionation assay (Additional file 1: Figure S4B and C).

Not surprisingly, we found that SPOP-ΔNLS was able to immunoprecipitate more endogenous Caprin1 than WT SPOP (Additional file 1: Figure S4D), and SPOP-ΔNLS was more effective at promoting Caprin1 degradation and ubiquitination than WT SPOP (Additional file 1: Figure S4E and F). These results suggest that SPOP can recruit Caprin1 into SPOP speckles to degradation, but this activity strictly depends on its cytoplasmic localization.

### SPOP suppresses Caprin1-dependent SG assembly

Caprin1 is required for SG assembly induced by oxidative stress [28]. Since SPOP controls Caprin1 protein abundance, we investigated whether SPOP can modulate SG assembly through Caprin1. First, we treated SPOP knockout and the corresponding parental C4–2 cells with sodium arsenite (AS), a classical SG inducer eliciting oxidative stress and scored them for SG assembly using eIF4G staining as a surrogate. As shown in Additional file 1: Figure S5, the levels of AS-induced SGs in SPOP knockout C4–2 cells were much higher than the levels in the control cells. Docetaxel, the most commonly-used therapeutic drug in prostate cancer, induced much more SGs in SPOP knockdout cells but less SGs in Caprin1 knocout cells compated with that in control cells (Additional file 1: Figure S6).

**Fig. 4:**
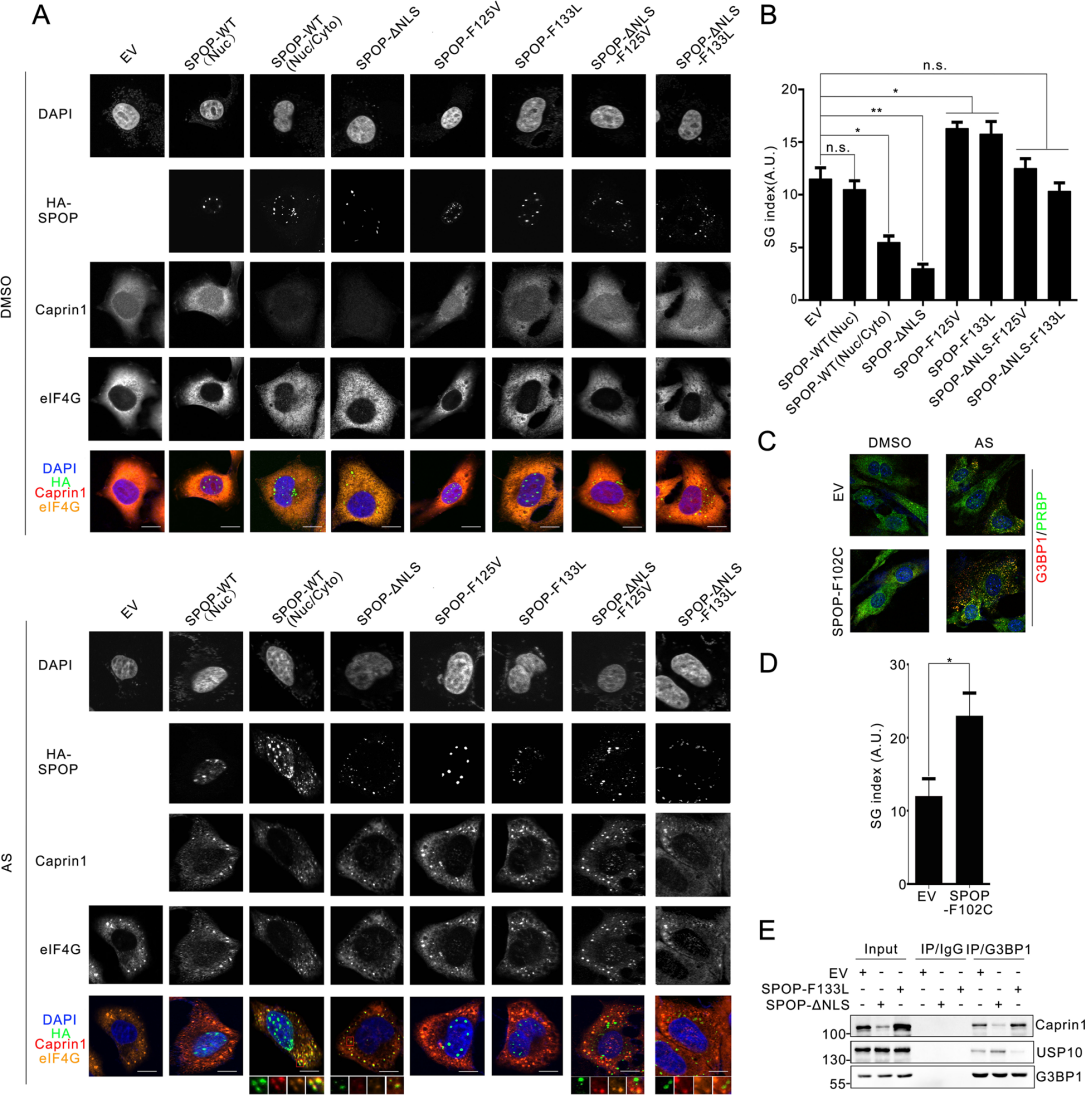
Wild-type SPOP suppresses, while the prostate cancer-associated mutants of SPOP enhances stress granules formation. **a** Representative immunofluorescence images of C4–2 cells transfected with indicated plasmids, treated with DMSO (*upper panel*) or AS (100 μM, 2 h) (*lower panel*) stained with SPOP(HA), Caprin1, EIF4G and DAPI. Scale bar, 20 μm. **b** SGs in (**a**) were quantified by defining a SG index (SG area/cell area) based on eIF4G immunofluorescence. Data are presented as arbitrary units (A.U.). Data are shown as means ± SD (n = 3) in which about 10 cells were quantified. **c** Representative immunofluorescence images of SPOP ^F102C^ MEFs, treated with AS (100 μM, 2 h), stained with G3BP1 and PABP. Scale bar, 20 μm. **d** SGs in (**c**) were quantified based on G3BP1 immunofluorescence. (**e** Western blot of indicated proteins in WCLs and co-IP samples of IgG or anti-G3BP1 antibody obtained from the cell extracts of C4–2 cells stably overexpressing EV, SPOP-ΔNLS or SPOP-F133 L

Ectopic expression of SPOP-ΔNLS in C4–2 cells significantly impaired AS-induced SG assembly compared with empty vector (EV)-transfected cells (Fig. 4a, b). Similar results were observed in SPOP-WT expressing cells when SPOP displayed cytoplasmic/nuclear localization, but not in cells where SPOP were exclusively localized in nuclei, suggesting SPOP-mediated suppression of SG assembly strictly depends on its cytoplasmic localization (Fig. 4a, b). In contrast, forced expression of prostate cancer-associated mutants of SPOP moderately increased SG assembly, probably by acting through a dominant-negative effect to inhibit endogenous SPOP (Fig. 4a, b). Although SPOP-F125 V-ΔNLS and F133 L-ΔNLS mutants accumulated exclusively in the cytoplasm in punctate patterns similar to SPOP-ΔNLS, they had no impact on AS-induced SG assembly (Fig. 4a, b). This result is expected, as these two mutants are unable to bind and degrade Caprin1 in the cytoplasm (Additional file 1: Figure S4E and F). We extended our analysis using MEFs derived from SPOP^F102C^-conditional knockin mice (Huang H, unpublished data). Expression of knockin SPOP-F102C mutant was achieved by infecting MEFs with lentivirus expressing CMV-driven Cre recombinase. As shown in Fig. 4c and d, SPOP-F102C mutant knockin caused MEFs to produce more SGs than EV-infected MEFs upon AS treatment. Taken together, these data demonstrate that WT SPOP suppresses SG assembly, while the prostate cancer-associated SPOP mutants enhances SG assembly.

Previous studies have demonstrated that Caprin1 binding to G3BP1 promotes SG formation, whereas USP10 inhibits SG assembly by competing with Caprin1 for the same binding site in G3BP1 [24]. As expected, expression of SPOP-ΔNLS mutant reduced Caprin1 level and increased G3BP1**–**USP10 interaction, while SPOP-F133 L mutant expressioin increased Caprin1 level and reduced G3BP1**–**USP10 interaction (Fig. 4e). To determine whether the suppressive role of SPOP in SG assembly is dependent on Caprin1 but not G3BP1, we generated a Caprin1 knockout cell line using CRISPR/Cas9 methods. Western blot analysis showed that Caprin1 protein expression was ablated (Additional file 1: Figure S2E-H). A previous study demonstrated that G3BP1, but not Caprin1, was required for clotrimazole (CZ)-induced SGs. We showed that SPOP-ΔNLS had no impact on CZ-induced SGs (Additional file 1: Figure S**7**A and B). Moreover, SPOP-ΔNLS was not co-localized with CZ-induced SGs in Caprin1 knockout cells (Additional file 1: Figure S**7**A), suggesting that SPOP suppresses SG assembly in a Caprin1-dependent manner.

We next asked whether SPOP is involved in stress-induced translational arrest. SPOP-ΔNLS-expressing or SPOP-depleted C4–2 cells were treated with the stress inducer AS, pulse-labeled with puromycin, and detected by western blot. AS treatment inhibited translation equally well in control and SPOP overexpressing/depleted cells, as shown by reduced ribopuromycin incorporation (Additional file 1: Figure S8A and B). Similarly, stress-induced phosphorylation of eIF2α was identical in control and SPOP-ΔNLS overexpressing/depleted cells, indicating that SPOP-suppressed SG assembly does not occur through modulation of the translational arrest process (Additional file 1: Figure S8A and B). We further asked whether SPOP prevents SG assembly by inhibiting polysome disassembly. As shown in Additional file 1: Figure S8C and D, SPOP-ΔNLS expression or SPOP depletion did not alter stress-induced polysome disassembly. These results indicate that SPOP suppresses SG assembly by preventing mRNP condensation through Caprin1 destruction, but has no impact on stress-induced translational arrest or polysome disassembly. mRNAs that are not engaged in translation can also aggregate into processing bodies (P-bodies), in addition to SGs [23]. We found that SPOP-WT or SPOP-ΔNLS were not co-localized with EDC4, a known component of P-bodies and regulator of mRNA decapping (Additional file 1: Figure S9). Moreover, SPOP expression had no impact on P-body assembly as evaluated by EDC4 puncta (Additional file 1: Figure S9). Thus, these results suggest that SPOP specifically regulates the assembly of Caprin1-dependent SGs, but not P-bodies.

### Prostate cancer-associated SPOP mutations enhance cancer cell survival through upregulating Caprin1

**Fig. 5:**
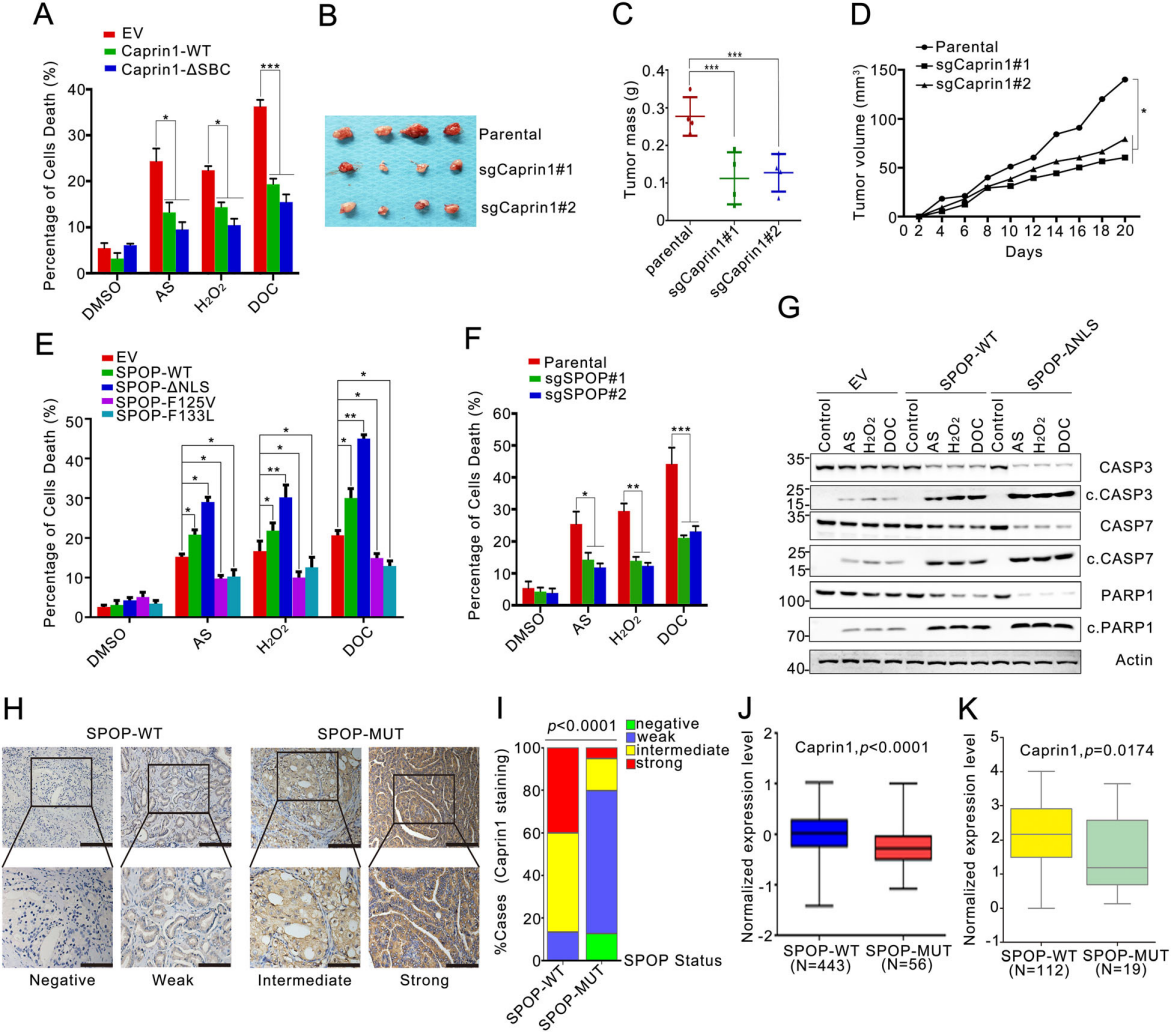
WT SPOP promotes, while the prostate cancer-associated SPOP mutants inhibit stress-induced cell death. **a** The cell death analysis (PI staining) of C4–2 cells stably expressing EV, SPOP-WT, ΔNLS, F125 V, or F133 L treated with DMSO, AS (100 μM, 2 h), H_2_O_2_(2 h)or Docetaxel (DOC, 10 μM, 4 h) .**b** The tumor image of parental and Caprin1 knockout C4–2 cells implanted tumors from nude mice. **c**, **d** Weight (**c**) and growth curve (**d**) and of implanted tumors formed by parental and Caprin1 knockout C4–2 cells. *n* = 4. **e** The cell death analysis of parental and Caprin1 knockout C4–2 cells treated with DMSO, AS (100 μM, 2 h), H_2_O_2_ (2 h) or DOC (10 μM, 12 h). **f** The cell death analysis of C4–2 cells stably expressing EV, Caprin1-WT, or ΔSBC treated with DMSO, AS (100 μM, 2 h), H_2_O_2_(2 h)or DOC (10 μM, 12 h) . **g** Western blot of WCLs from C4–2 cells stably expressing EV, SPOP-WT, or SPOP-ΔNLS treated with DMSO, AS (100 μM, 2 h), H_2_O_2_(2 h)or DOC (10 μM, 12 h) . **h** Representative IHC images of Caprin1 staining in SPOP wild-type or mutant primary human prostate cancer samples. The scale bar: 400 μm or 100 μm. **i** Quantitative data for the Caprin1 protein staining in (**g**). Statistical significance was determined by Wilcoxon rank-sum test. **j** RT-qPCR assessment of Caprin1 mRNA expression in SPOP-WT and SPOP-MUT prostate tumors. Caprin1 mRNA expression level in each tumor specimen was normalized by the expression level of 18S rRNA (internal control) and exhibited as a value. of log^10^. *P* values were determined by Mann-Whitney test (two-sided). **k** Comparing Caprin1 mRNA expression between SPOP-WT and SPOP-MUT prostate tumors using TCGA RNA-seq data. Y-axis indicates the mean-centered gene expression level pre calculated from pan-cancer analysis. *P* values were determined by non-parametric Wilcoxon rank sum test (two sided)

Recently, emerging evidences support a positive role for SGs in tumor cell fitness in various cancer models [25, 26]. To determine the biological importance of SPOP regulation of Caprin1-mediated SG assembly in prostate cancer, we first investigated whether Caprin1 expression is important for maintaining the neoplastic phenotypes of prostate cancer cells. However, knockout or overexpression of Caprin1 alone had no obvious effect on the DNA synthesis (Additional file 1: Figure S10A and B), cell cycle progression (Additional file 1: Fig. S10C and D), cell growth (Additional file 1: Figure S10E and F) and migration (Additional file 1: Figure S10G and H) in prostate cancer cells. Because SG assembly is a conserved strategy utilized by cells to minimize stress-induced damage and promote cell survival under unfavorable circumstances, we investigated whether the Caprin1 expression level is closely related to cell survival upon treatment with AS, H_2_O_2_ and the chemotherapeutic drug docetaxel (DOC), all of which are potent SG inducers. Caprin1 overexpression protected cancer cells from AS, H_2_O_2_ or DOC-induced cell death as measured by propidium iodide staining (Fig. 5a), while Caprin1 knockout sensitized cancer cells to stress-induced cell death (Additional file 1: Figure S10I). Moreover, knockout of Caprin1 in C4–2 cells significantly reduced the growth of tumor xenografts in mouse models **(**Fig. 5b-d). These results suggest that Caprin1 is an important cell survival regulator under stress conditions in vitro and in vivo. Moreover, expression of SPOP-WT or the ΔNLS mutant significantly increased stress-induced cell death, while expression of prostate cancer-associated SPOP mutants (F125 V, F133 L) or knockout of endogenous SPOP by shRNAs suppressed stress-induced cell death **(**Fig. 5e, f). Furthermore, a non-SPOP-degradable Caprin1-ΔSBC mutant resulted in more protective effect against stress-induced cell death than WT Caprin1 **(**Fig. 5a). Finally, WB analysis demonstrated that expression of SPOP-WT or the ΔNLS mutant enhanced stress-induced cell apoptosis, as evidenced by caspase 3/7 and PARP1 cleavages **(**Fig. 5g). Taken together, these results suggested that the SPOP-Caprin1 regulatory axis might be critical for cell survival under environmental stress in prostate cancer cells.

To examine the effect of SPOP mutations on Caprin1 protein levels in prostate cancer specimens from patients, we analyzed Caprin1 protein levels by immunohistochemistry (IHC) methods in a cohort for which a total of 131 primary prostate tumor samples were available (Additional file 2: Table S2). The antibody specificity for IHC analysis was validated in parental/Caprin1 knockout cell lines (Additional file 1: Figure S11). A total 19 of SPOP-mutant tumors were identified through Sanger sequencing. IHC analysis showed that approximately 80% of SPOP-mutated tumors exhibited strong or intermediate staining for Caprin1 (Fig. 5g, h). In contrast, approximately 20% of tumors with WT SPOP exhibited strong or intermediate staining for Caprin1 and the majority of the tumors with WT SPOP (approximately 60%) exhibited weak staining (Fig. 5g, h). Expression of Caprin1 mRNA was roughly equal between SPOP-mutated/SPOP-WT tumors as measured by RT-qPCR (Fig. 5i). The Cancer Genome Atlas (TCGA) dataset showed that Caprin1 mRNA level were even lower in SPOP-mutated tumors than in specimens with WT SPOP (Fig. 5j). Interstinlgy, we found a statistically significant positive correlation between Caprin1 IHC intensity and preoperative serum PSA level, but not the Gleason score and pathologic T stage (Additional file 2: Table S3). As PSA is a strong predictor of prostate cancer prognosis, it can be inferred that Caprin1 protein level might be associated with the aggressiveness of prostate cancer [29]. Collectively, these data suggest that SPOP mutations result in elevated Caprin1 protein abundance that associated with prostate cancer progression.

## Discussion

**Fig. 6:**
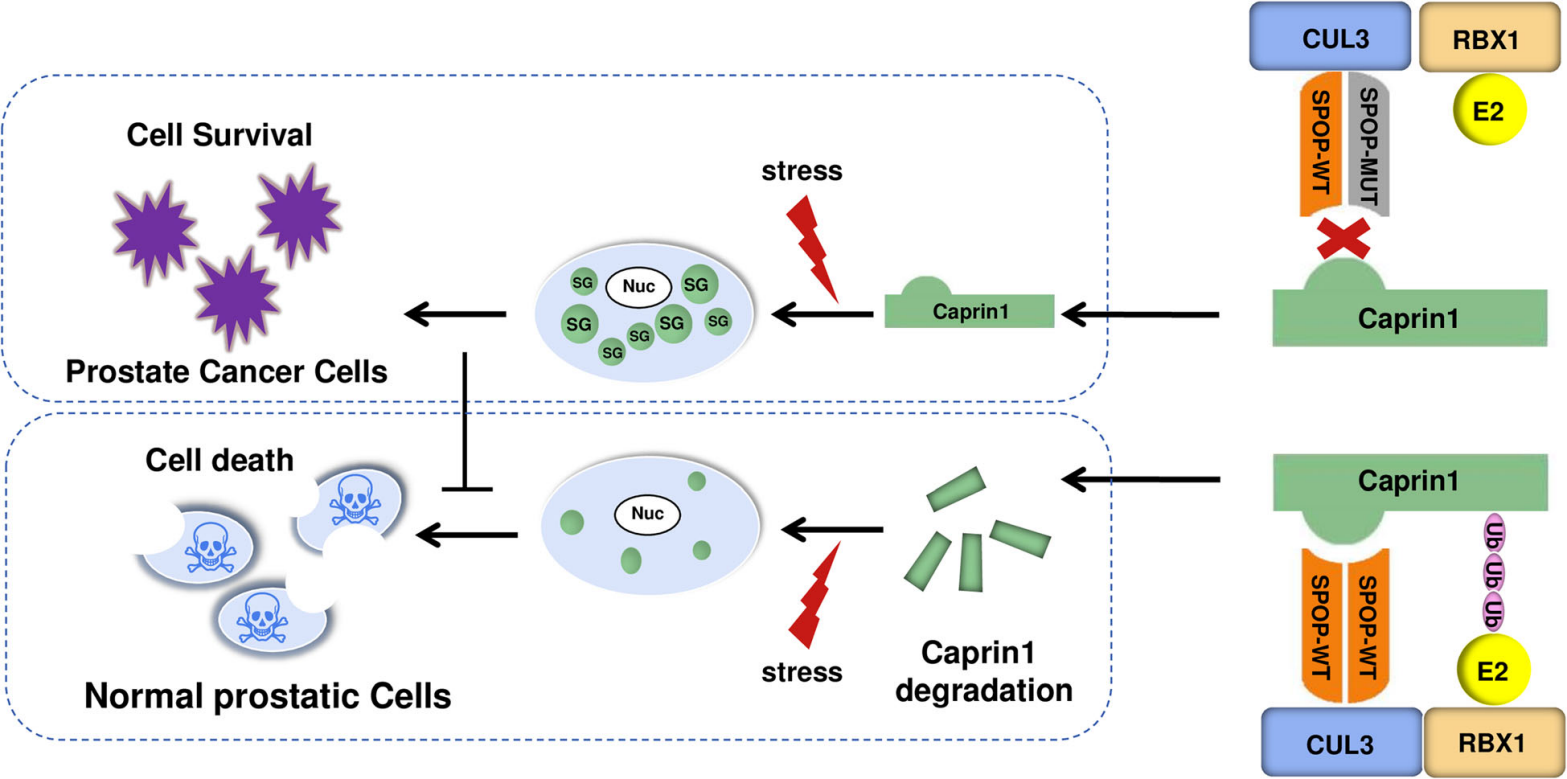
Schematic of the proposed mechanism through which SPOP mutants enhance SG assembly and resistance to stress-induced cell death in SPOP-mutated prostate cancer

Increasing evidence indicates that SPOP mutated prostate cancer has a unique molecular and phenotypical features. However, causal mechanisms and signaling are not fully understood. Previous studies have showed that SPOP inactivation increased prostate cell proliferation, migration and invasion in both an AR-dependent and independent manner [10, 11]. Moreover, SPOP inactivation leads to compromised anti-tumor immunity by increasing PD-L1 protein stability in cancer cells [16]. In the current study, we revealed that Caprin1 is a bona fide SPOP substrate. Caprin1 plays minimal roles in prostate cancer proliferation and migration in vitro, but is important for cell survival under stress conditions and tumor growth in vivo. WT SPOP suppresses SG assembly and promotes stress-induced cell death in prostate cancer cells by targeting Caprin1 for ubiquitin-dependent degradation, and this effect is abrogated by prostate cancer-associated SPOP mutations (Fig. 6). Collectively, we identified a novel SPOP mutation-driven protumorigenic process in prostate cancer by upregulating SG assembly. Interference with this pathway may have therapeutic benefit by re-sensitizing mutant SPOP cells to chemotherapeutic agents, such as docetaxel.

The identification of specific genetic alterations that drive the initiation and progression of cancer, and the development of targeted drugs that act against these driving alterations, have revolutionized the treatment of many human cancers. During the last decade, evidence has accumulated that SGs play important roles in regulating cancer initiation, progression, metastasis and therapeutic resistance [22, 30]. Inhibition of SG assembly is expected to make cancer cells vulnerable to therapeutic drugs [22]. However, the underlying mechanism by which genetic alternations lead to aberrant SG assembly in cancer cells is still poorly understood. A previous study demonstrated that SG assembly is elevated in mutant K-RAS pancreatic cancer cells in response to a variety of stress stimuli [31]. The upregulation of SGs mediated by mutant K-RAS-dependent pathways is dependent on the production of the signaling lipid molecule 15-d-PGJ2 and confers cytoprotection against stress stimuli and chemotherapeutic agents [31]. It will be interesting to explore whether upregulation of SG assembly is a universal phenomenon in various subtypes of prostate cancer or unique to the SPOP-mutated subtype, and whether other frequently mutated genes in prostate cancer, such as *PTEN, FOXA1, PIK3CA, MED12,* and *TP53*, are also involved in regulation of SG assembly. In addition to Caprin1, G3BP2 expression is an independent prognostic factor predicting poor outcome in prostate cancer [32]. Further studies are warranted to clarify how genetic alteration alters the expression level or the post-translational modification of SG nucleating proteins in prostate cancer.

## Conclusion

During recent years, significant progress has been made in identifying the molecular alterations in prostate cancer through next-generation sequencing. The *SPOP* gene is the most frequently mutated gene in primary prostate cancer. However, the precise role of SPOP mutations in prostate oncogenesis remains largely unclear. Chemotherapy drugs such as docetaxel can induce SG formation, which mitigates the cell-killing effects of the drugs. Resistance to docetaxel is a major clinical problem in advanced prostate cancer. Our study reveals that SPOP mutations augment Caprin1-mediated SG assembly and render prostate cancer cells resistance to docetaxel, suggesting that targeting the SG assembly pathway represents a viable strategy to restore docetaxel sensitivity in prostate cancer cells.

## Supplementary information

**Additional file 1: Figure S1.** The mRNA and protein expression of SPOP/Caprin1 in prostate cancer cells. **Figure S2.** Validation of SPOP/Caprin1 knockout in C4–2 cells. **Figure S3.** The SBC motif in Caprin1 is a degron recognized by SPOP. **Figure S4.** SPOP-ΔNLS mutant is constitutively localized in cytoplasm as puncta and more potent in promote Caprin1 degradation than wildtype SPOP-WT. **Figure S5.** SPOP knockout enhanced AS-induced SGs assembly in C4–2 cells. **Figure S6.** SPOP knockout enhances, while Carpin1 knockout suppresses Docetaxel-induced stress granules assembly in C4–2 cells. **Figure S7.** SPOP had no impact on clotrimazole-induced SG assembly. **Figure S8.** SPOP is dispensable for stress-induced translational arrest. **Figure S9.** SPOP had no impact on P-bodies assembly. **Figure S10.** Knockout or overexpression of Caprin1 marginally affected the growth or migration, but significantly increased stress-induced cell death in C4–2 cells. **Figure S11.** Validation of anti-Caprin1 antibody for IHC through using parental and Caprin1 knockout cells.

**Additional file 2: Table S1.** Primers, sequences of shRNAs and siRNAs, antibody and chemicals. **Table S2.** SPOP mutation status, Caprin1 IHC scores in 131 cases of prostate cancer specimens and the associated clinical information. Table S3. Primers, sequences of shRNAs and siRNAs, antibody and chemicals.

## Data Availability

The data used or analyzed during this study are included in this article and available from the corresponding author upon reasonable request.

## Abbreviations

AS: Sodium arsenite
ATCC: American Type Culture Collection
CCK-8: Cell Counting Kit-8
CZ: Clotrimazole
FFPE: Formalin-fixed paraffin-embedded
IHC: Immunohistochemistry
IP: Immunoprecipitation
IP: Immunoprecipitation
MEF: Mouse embryonic fibroblasts
mRNPs: Messenger ribonucleoprotein particles
RT-qPCR: Quantitative real-time polymerase chain reaction
SG: Stress granules
shRNA: Short hairpin RNA
siRNA: Small interfering RNA
SPOP: Speckle type BTB/POZ protein
WT: Wild-Type; Fibroblasts
