# Supplementary material for: Prostate Cancer-associated SPOP mutations enhance cancer cell survival and docetaxel resistance by upregulating Caprin1-dependent stress granule assembly
Source: Mol Cancer. 2019 Nov 26;18:170. doi: 10.1186/s12943-019-1096-x (PMC6878651; doi:10.1186/s12943-019-1096-x)
Supplement: Supplementary file 1 — Additional file 1: Figure S1. The mRNA and protein expression of SPOP/Caprin1 in prostate cancer cells. Figure S2. Validation of SPOP/Caprin1 knockout in C4–2 cells. Figure S3. The SBC motif in Caprin1 is a degron recognized by SPOP. Figure S4. SPOP-ΔNLS mutant is constitutively localized in cytoplasm as puncta and more potent in promote Caprin1 degradation than wildtype SPOP-WT. Figure S5. SPOP knockout enhanced AS-induced SGs assembly in C4–2 cells. Figure S6. SPOP knockout enhances, while Carpin1 knockout suppresses Docetaxel-induced stress granules assembly in C4–2 cells. Figure S7. SPOP had no impact on clotrimazole-induced SG assembly. Figure S8. SPOP is dispensable for stress-induced translational arrest. Figure S9. SPOP had no impact on P-bodies assembly. Figure S10. Knockout or overexpression of Caprin1 marginally affected the growth or migration, but significantly increased stress-induced cell death in C4–2 cells. Figure S11. Validation of anti-Caprin1 antibody for IHC through using parental and Caprin1 knockout cells. [file 12943_2019_1096_MOESM1_ESM.docx]

**Additional file 1 Legends**

**
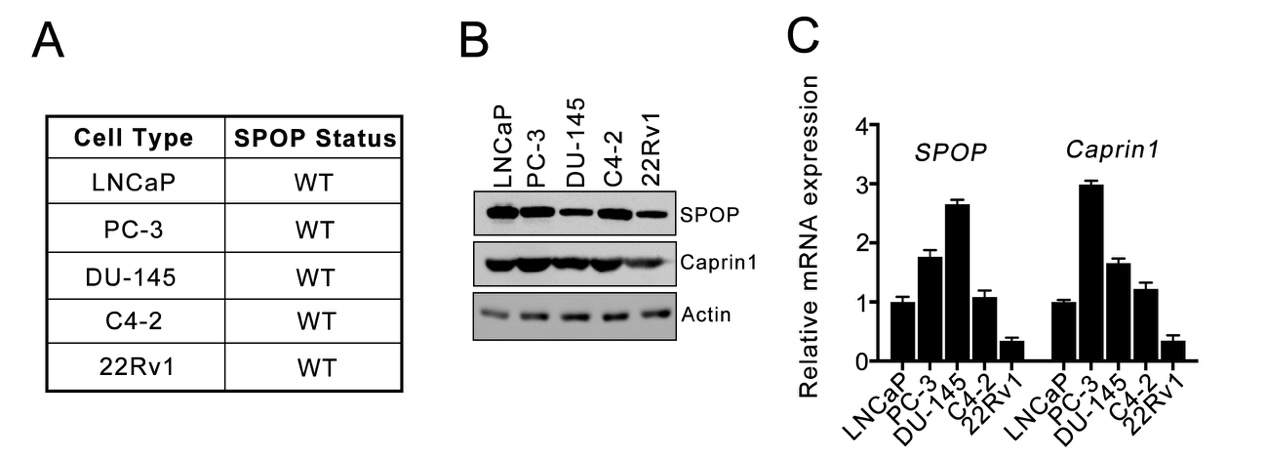
**

**Supplementary Figure. 1** **The mRNA and protein expression of SPOP/Caprin1 in prostate cancer cells.**

(A) The SPOP mutation status of prostate cancer cell lines used in this study. The mutation information was obtained from Cancer Cell Line Encyclopedia (https://portals.broadinstitute.org/ccle) and previous literature.

(B) Western blot of the indicated proteins in WCLs from multiple prostate cancer cell lines.

(C) RT-qPCR assessment of SPOP/Caprin1 mRNA expression in multiple prostate cancer cell lines. The mRNA level of GAPDH was used for normalization. Data are shown as means ± SD (n=3).


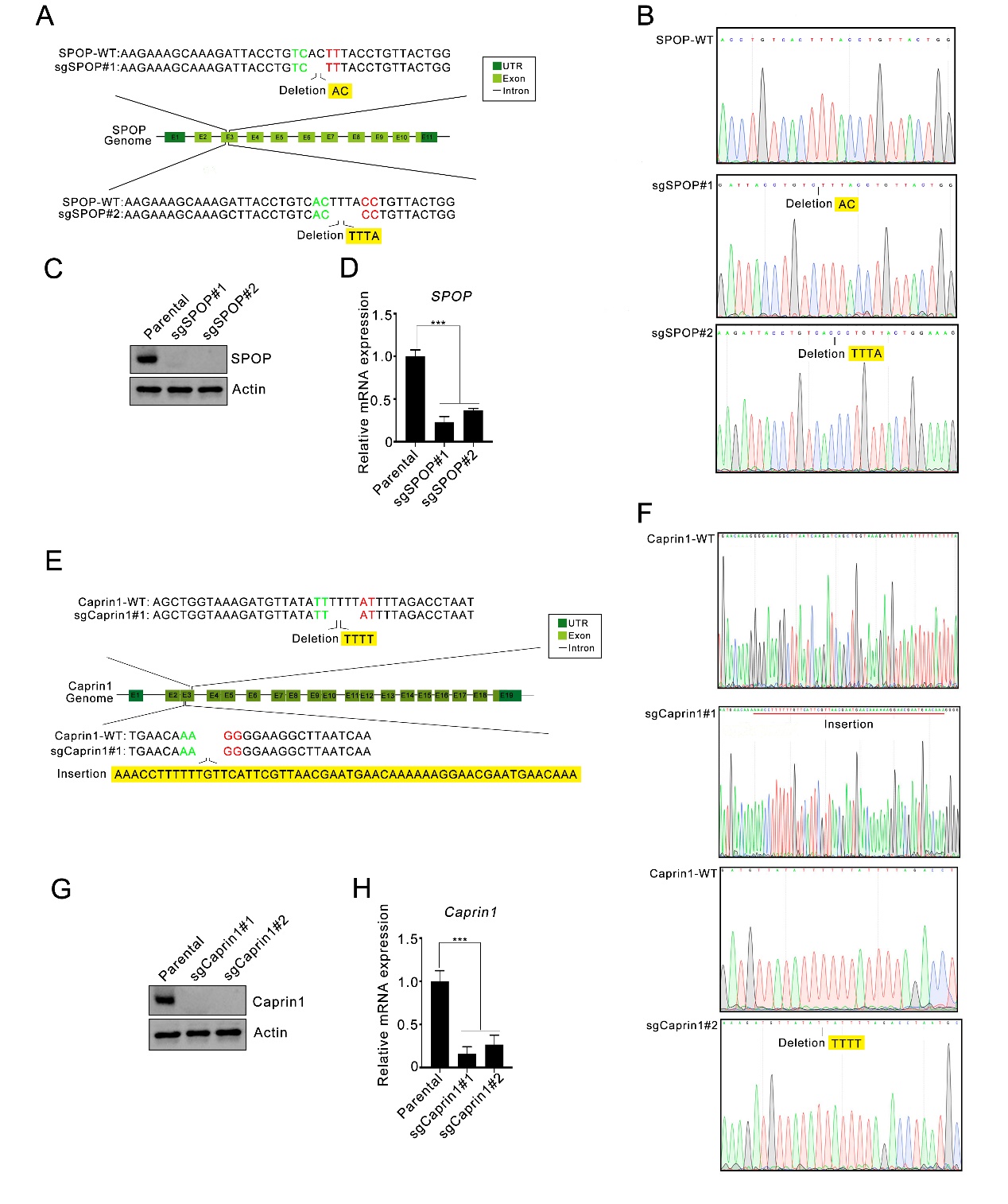


**Supplementary Figure. 2** **Validation of SPOP/Caprin1 knockout in C4-2 cells.**

(A) Schematic of CRISPR/Cas9-mediated knockout of SPOP in C4-2 cells.

(B) Sanger sequencing confirming that SPOP gene was edited in SPOP-KO C4-2 cells.

(C) Western blot of the indicated proteins in WCLs from parental and SPOP-KO C4-2 cells.

(D) RT-qPCR assessment of SPOP mRNA expression parental and SPOP-KO C4-2 cells.

The mRNA level of GAPDH was used for normalization. Data are shown as means ± SD (n=3). ***p<0.001.

(E) Schematic of CRISPR/Cas9-mediated knockout of Caprin1 in C4-2 cells.

(F) Sanger sequencing confirming that Caprin1 gene was edited in SPOP-KO C4-2 cells.

(G) Western blot of the indicated proteins in WCLs from parental and Caprin1-KO C4-2 cells. (H) RT-qPCR assessment of Caprin1 mRNA expression parental and Caprin1-KO C4-2 cells.

The mRNA level of GAPDH was used for normalization. Data are shown as means ± SD (n=3). ***p<0.001.

**
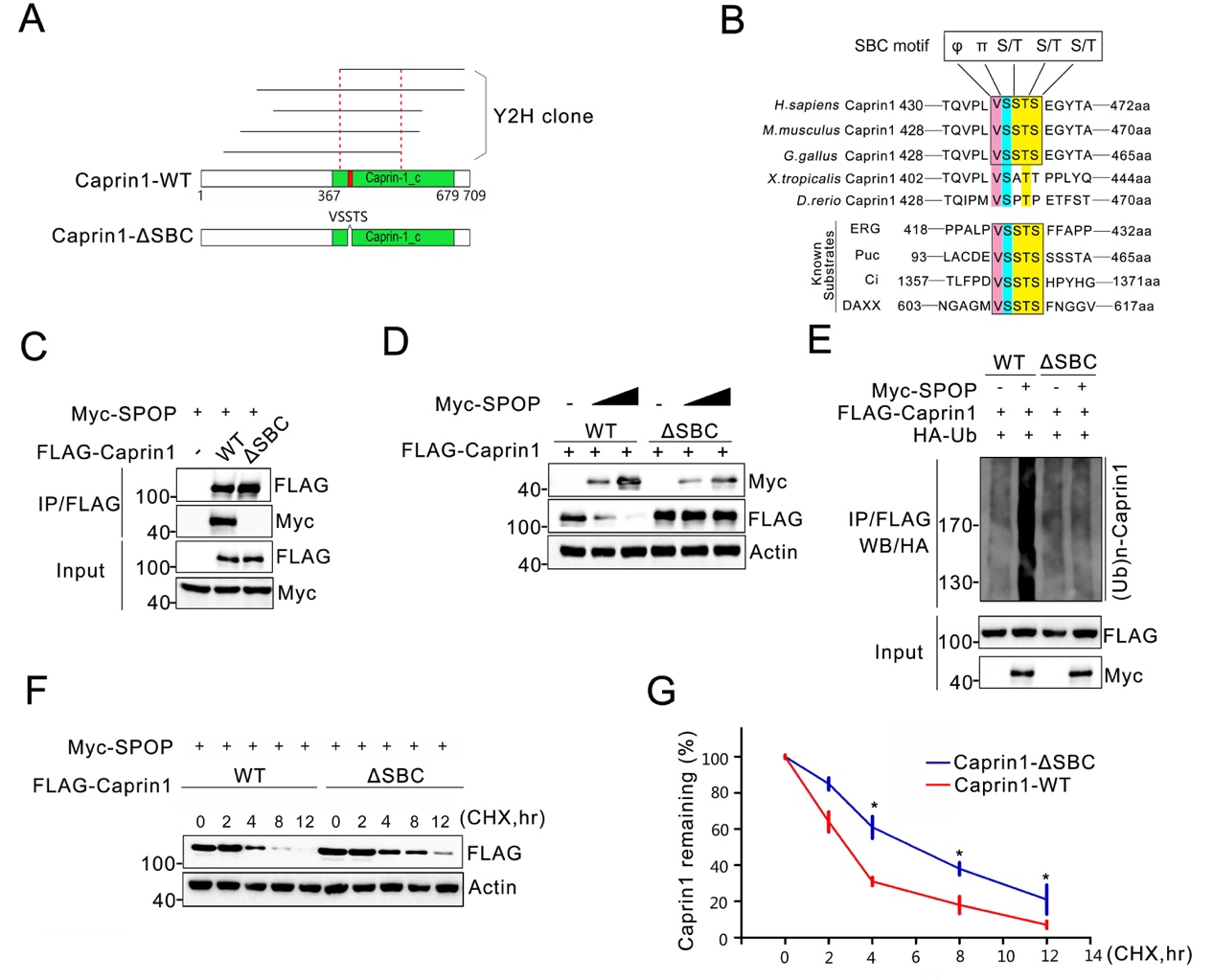
**

**Supplementary Figure. 3** **The SBC motif in Caprin1 is a degron recognized by SPOP.**

(A) Diagram showing the portions of Caprin1 identified by yeast two-hybrid (Y2H) screen in a human fetal brain cDNA library using full-length SPOP as bait. The region between each pair of dashed red lines is the minimal interaction region shared by positive clones, and the red rectangles represent the SBC motif.

(B) Amino acid sequence alignment of putative SBC motifs in Caprin1. ERG, Puc, Ci and Daxx are known SPOP substrates containing well-characterized SBC motifs. (Φ-π-S-S/T-S/T; Φ: nonpolar residues, π: polar residues)

(C) Western blot of indicated proteins in WCLs and samples from co-IP with anti-FLAG antibody in 293T cells transfected with the indicated plasmids.

(D) Western blot of indicated proteins in WCLs from 293T cells transfected with the indicated plasmids.

(E) Western blot of the products of in vivo ubiquitination assays from 293T cells transfected with the indicated plasmids and treated with 20 µM MG132 for 8 hr.

(F, G) Western blot of indicated proteins in WCLs from 293T cells transfected with the indicated plasmids, treated with 50 µg/ml cycloheximide (CHX) and harvested at different time points (F). At each time point, the intensity of Caprin1 was normalized to the intensity of actin and then to the value at 0 hr (G). The experiment was done in triplicates. Data are shown as means ± SD (n=3). *p<0.05.

**
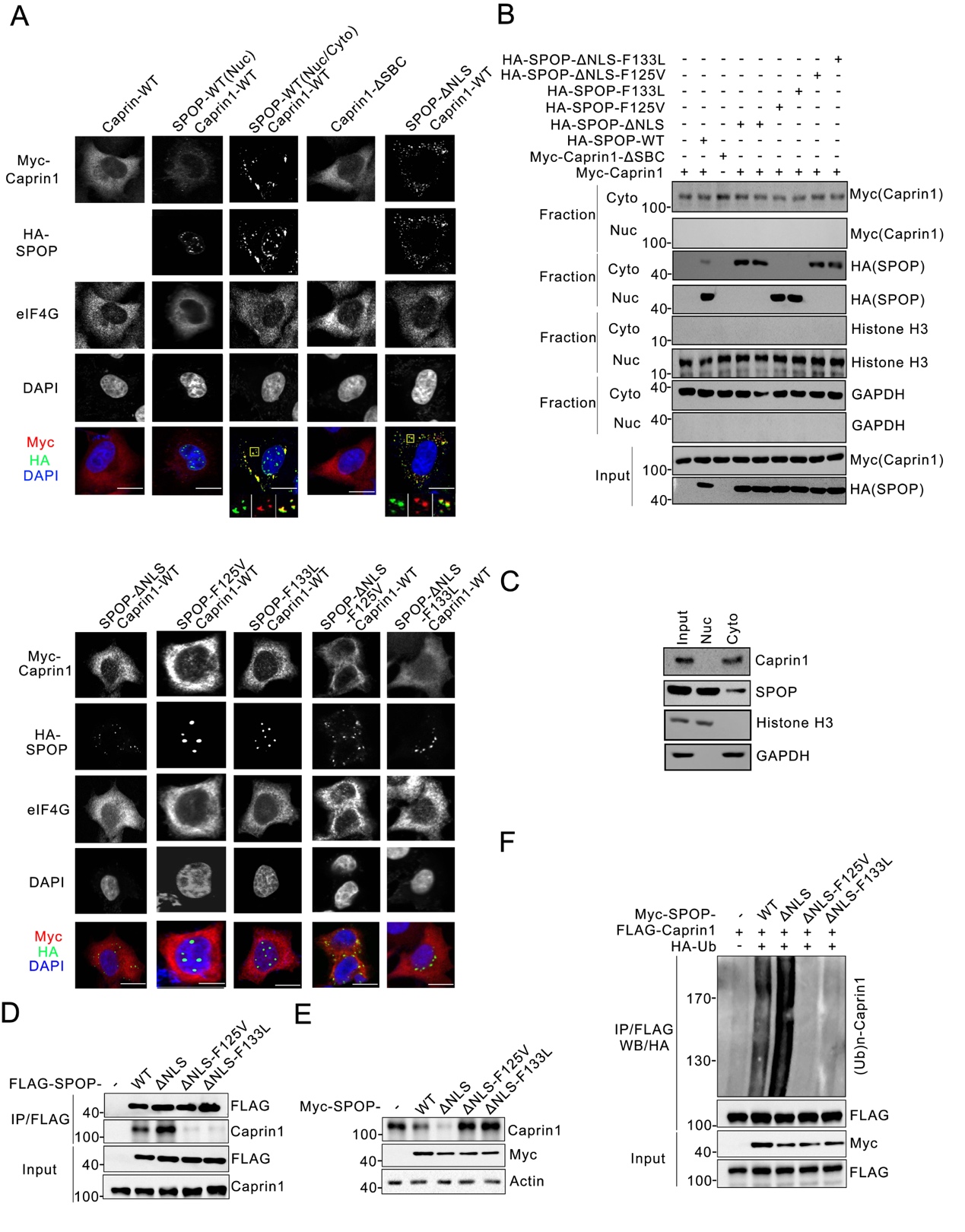
**

**Supplementary Figure. 4** **SPOP-ΔNLS mutant is constitutively localized in cytoplasm as puncta and more potent in promote Caprin1 degradation than wildtype SPOP-WT.**

(A) Representative immunofluorescence images of C4-2 cells transfected with indicated plasmids, stained with SPOP(HA), Caprin1 (Myc), eIF4G and DAPI. Scale bar, 20 μm.

(B) Western blot of cytoplasmic and nuclear extracts of SPOP/Caprin1-overexpressing HeLa cells with indicated antibodies.

(C) Western blot of cytoplasmic and nuclear extracts of C4-2 cells with indicated antibodies.

(D) Western blot of indicated proteins in WCLs and samples from co-IP with anti-FLAG antibody in 293T cells transfected with the indicated plasmids and treated with 20 µM MG132 for 8 hr.

(E) Western blot of indicated proteins in WCLs from 293T cells transfected with the indicated plasmids.

(F) Western blot of the products of in vivo ubiquitination assays from 293T cells transfected with the indicated plasmids and treated with 20 µM MG132 for 8 hr.

**
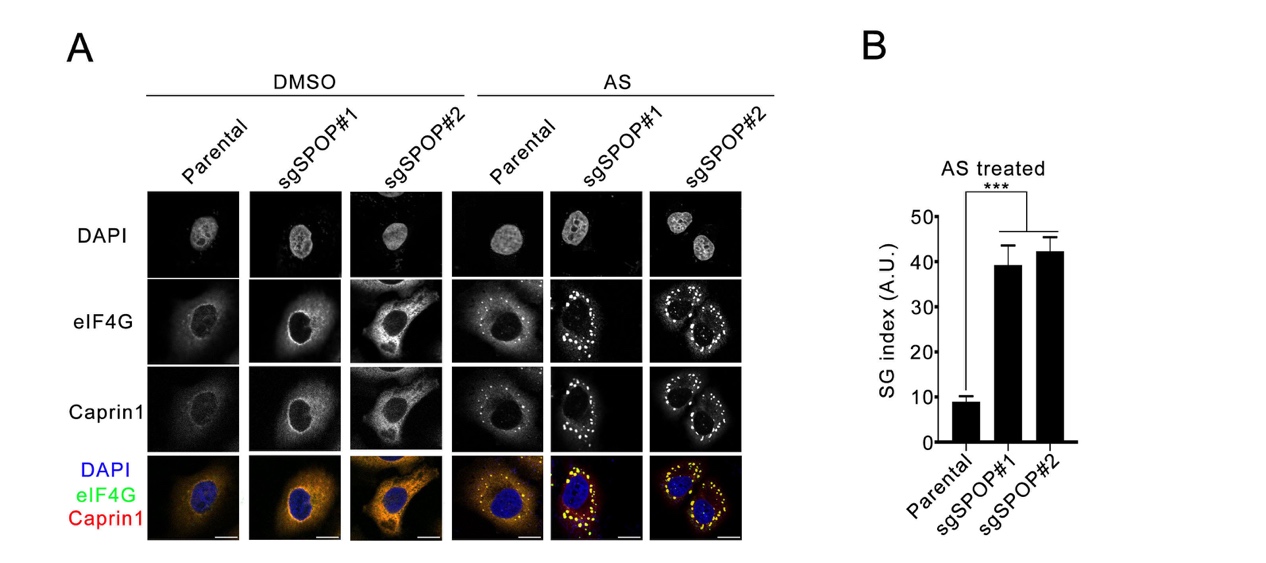
**

**Supplementary Figure. 5 SPOP knockout enhanced AS-induced SGs assembly in C4-2 cells.**

(A) Representative immunofluorescence images of parental and two SPOP knockout C4-2 cell clones treated with DMSO or arsenite sodium (100 μM, 2hr), stained with Caprin1, EIF4G and DAPI. Scale bar, 20 μm.

(B) SGs in (A) were quantified were quantified based on EIF4G immunofluorescence. ***p<0.001.


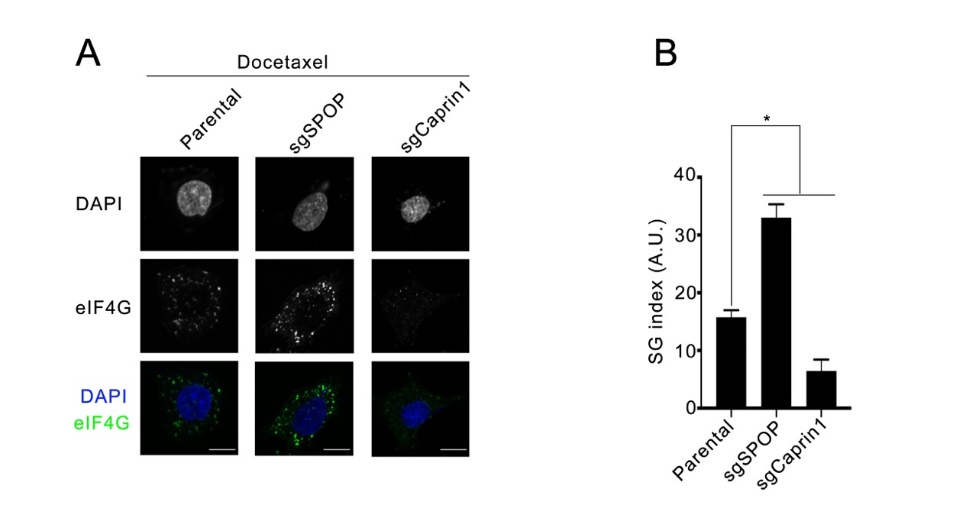


**Supplementary Figure. 6 SPOP knockout enhances, while Carpin1 knockout suppresses Docetaxel-induced stress granules assembly in C4-2 cells.**

(A) Representative immunofluorescence images of parental C4-2 cells or SPOP, Caprin1 knockout cells, treated with DOC (10 μM, 4hr), stained with EIF4G and DAPI. Scale bar, 20 μm.

(B) SGs in (A) were quantified based on EIF4G immunofluorescence. *p<0.05.


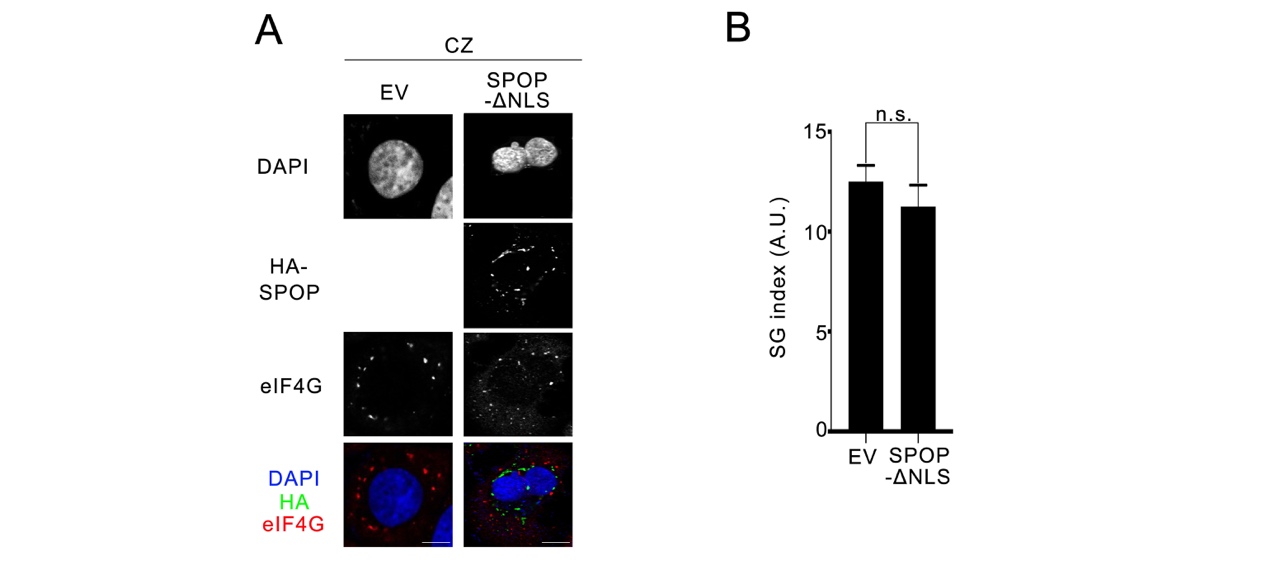


**Supplementary Figure. 7 SPOP had no impact on clotrimazole-induced SG assembly.**

(A) Representative immunofluorescence images of C4-2 cells transfected with HA-SPOP-ΔNLS, treated with arsenite sodium (100 μM, 2hr), stained with SPOP(HA), EIF4G and DAPI. Scale bar, 20 μm.

(B) SGs in (A) were quantified based on EIF4G immunofluorescence.


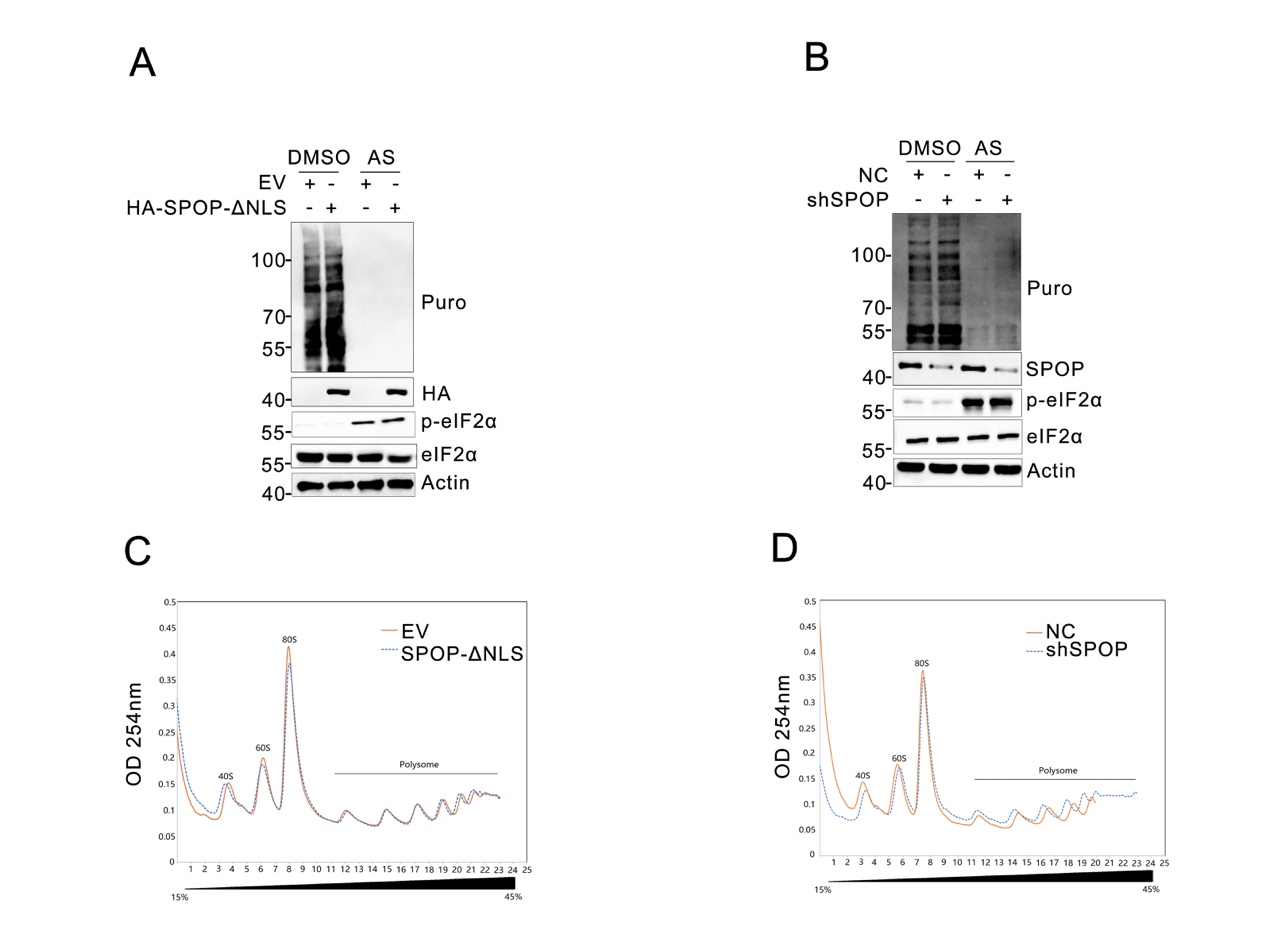


**Supplementary Figure. 8 SPOP is dispensable for stress-induced translational arrest.**

(A) Western blot of indicated proteins in WCLs from EV or HA- SPOP-ΔNLS stably expressing cells treated with DMSO or AS (100 µM, 2hr).

(B) Western blot of indicated proteins in WCLs from NC or SPOP shRNA expressing cells treated with DMSO or AS (100 µM, 2hr). _­_

(C) Polysome profiles obtained from cells as in (A).

(D) Polysome profiles obtained from cells as in (B).


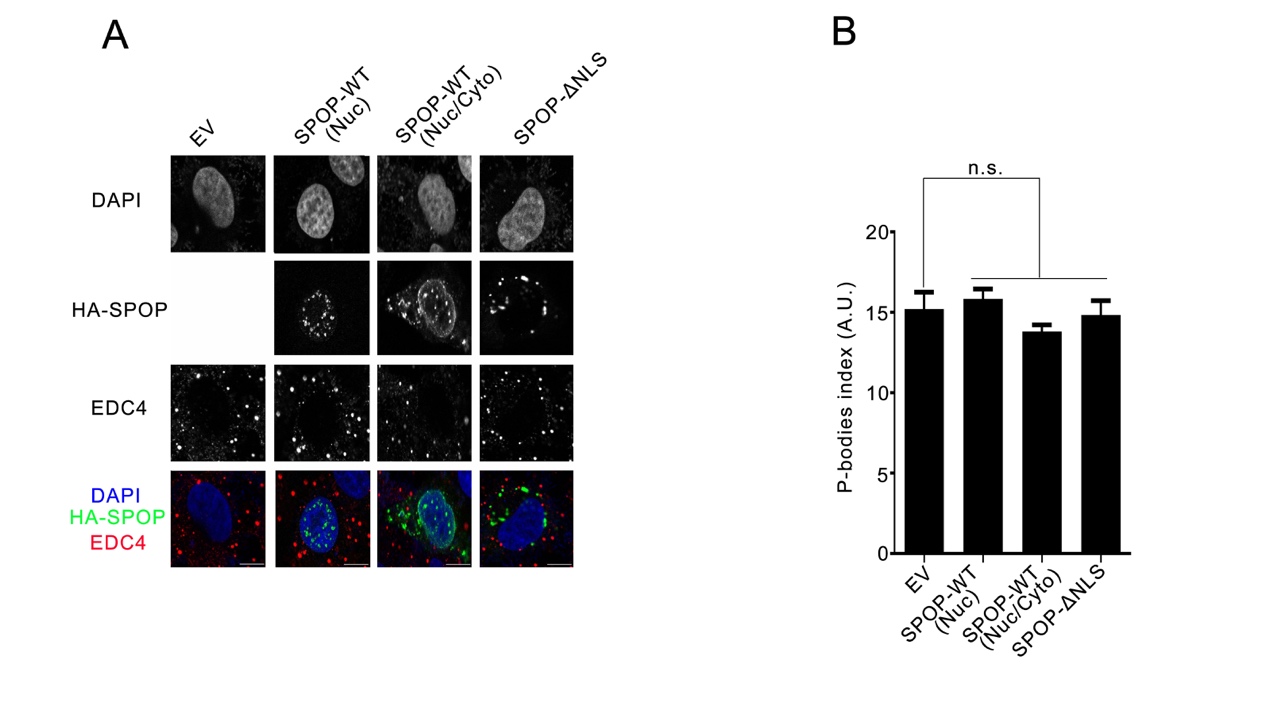


**Supplementary Figure 9. SPOP had no impact on P-bodies assembly.**

(A) Representative Immunofluorescence images of C4-2 cells transfected with HA-SPOP, stained with SPOP(HA), EDC4 and DAPI. Scale bar, 20 μm.

(B) SGs in (A) were quantified by defining a SG index (SG area/cell area) based on EDC4 immunofluorescence. Data are presented as arbitrary units (A.U.). Data are shown as means ± SD (n=3) in which about 10 cells were quantified.

_
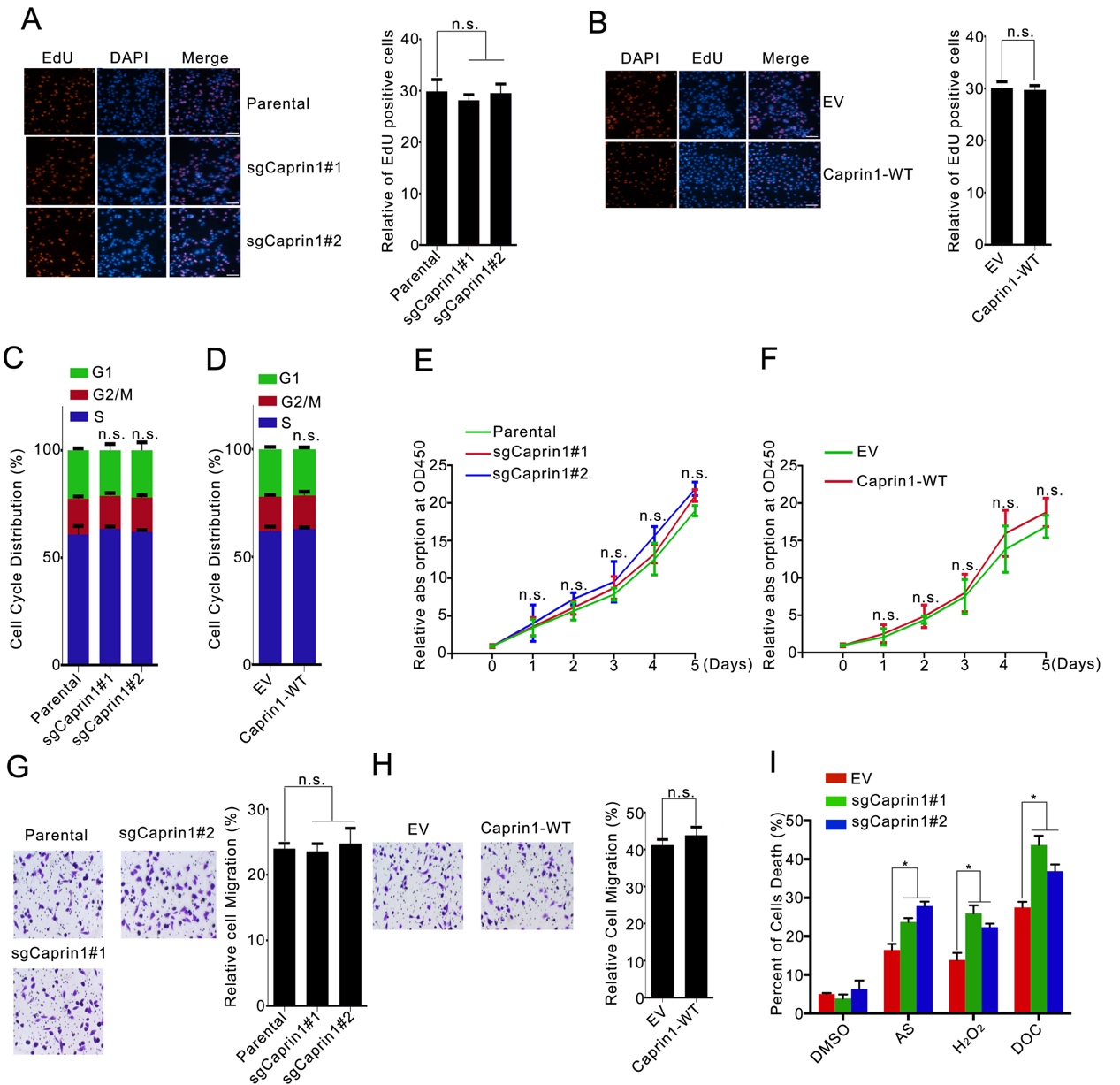
_

**Supplementary Figure 10. Knockout or overexpression of Caprin1 marginally affected the growth or migration, but significantly increased stress-induced cell death in C4-2 cells.**

(A) EdU incorporation analysis of parental or Caprin1 knockout C4-2 cells. and the quantitative analysis is shown on the right panel. Data are shown as means ± SD (n=3).

(B) EdU incorporation analysis of EV or Caprin1 stably overexpressing C4-2 cells. and the quantitative analysis is shown on the right panel. Data are shown as means ± SD (n=3).

(C) Cell cycle analysis of parental or Caprin1 knockout C4-2 cells. Data are shown as means ± SD (n=3).

(D) Cell cycle analysis of EV or Caprin1 stably overexpressing C4-2 cells. Data are shown as means ± SD (n=3).

(E) CCK8 cell proliferation analysis of parental or Caprin1 knockout C4-2 cells. Data are shown as means ± SD (n=3).

(F) CCK8 cell proliferation analysis of EV or Caprin1 stably overexpressing C4-2 cells. Data are shown as means ± SD (n=3).

(G) Cell migration analysis of parental or Caprin1 knockout C4-2 cells. and the quantitative analysis is shown on the right panel. Data are shown as means ± SD (n=3).

(H) Cell migration analysis of EV or Caprin1 stably overexpressing C4-2 cells. and the quantitative analysis is shown on the right panel. Data are shown as means ± SD (n=3).

(I) The cell death analysis of parental and two independent Caprin1 knockout C4-2 cells (#1,#2) treated with DMSO, AS (100 µM, 2hr)，H_2_O_2_（2hr）or DOC (10 µM, 12 hr). *p<0.05.


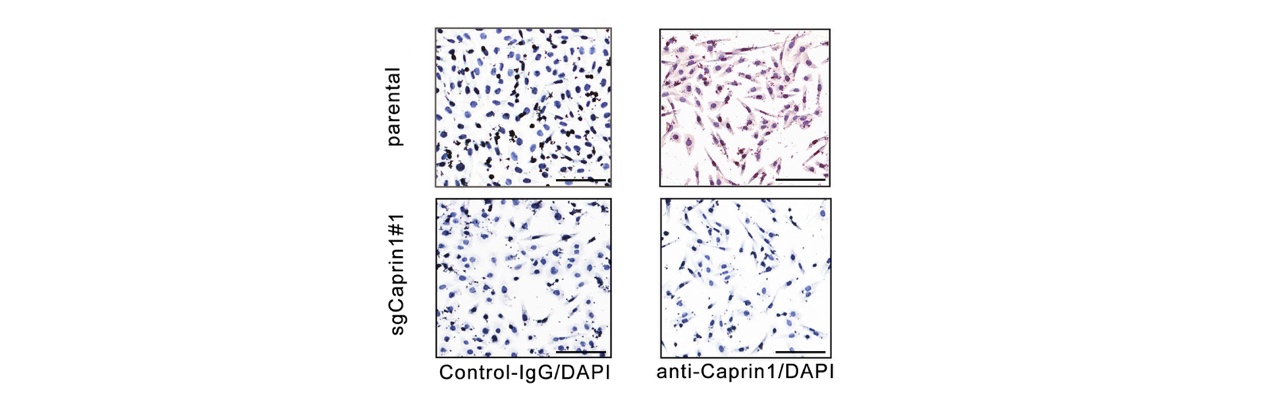


**Supplementary Figure 11. Validation of anti-Caprin1 antibody for IHC through using parental and Caprin1 knockout cells.**

Immunohistochemistry analysis of parental and Caprin1 knockout C4-2 cells from cultures on glass slides using the anti-Caprin1 antibody. Scale bar, 100 μm.
