# Supplementary material for: Prostate Cancer-associated SPOP mutations enhance cancer cell survival and docetaxel resistance by upregulating Caprin1-dependent stress granule assembly
Source: Mol Cancer. 2019 Nov 26;18:170. doi: 10.1186/s12943-019-1096-x (PMC6878651; doi:10.1186/s12943-019-1096-x)
Supplement: Supplementary file 2 — Additional file 2: Table S1. Primers, sequences of shRNAs and siRNAs, antibody and chemicals. Table S2. SPOP mutation status, Caprin1 IHC scores in 131 cases of prostate cancer specimens and the associated clinical information. Table S3. Primers, sequences of shRNAs and siRNAs, antibody and chemicals. [file 12943_2019_1096_MOESM2_ESM.docx]

**Additional file 2: Table S1. Primers , sequences of shRNAs and siRNAs, antibody and chemicals.**

|  | |  | | |  |  |  |
| --- | --- | --- | --- | --- | --- | --- | --- |
| **Primers for RT-qPCR with cell lines samples** | | | | | |  |  |
| Gene name | | F: 5'-3' | | | R: 5'-3' |  |  |
| SPOP | | TGAAGCCAGAGAGCGGTATGC | | | GATTGCTTCAGGCGTTTGCGTG |  |  |
| Caprin1 | | TCTCGGGGTGATCGACAAGAA | | | CCCTTTGTTCATTCGTTCCTGG |  |  |
| GAPDH | | TGCACCACCAACTGCTTAGC | | | GGCATGGACTGTGGTCATGAG |  |  |
| **Primers for RT-qPCR with FFPE patient tumor samples with FFPE patient tumor samples** | | | | | |  |  |
| Gene name | | F: 5'-3' | | | R: 5'-3' |  |  |
| Caprin1 | | TCTCGGGGTGATCGACAAGAA | | | CCCTTTGTTCATTCGTTCCTGG |  |  |
| 18s RNA | | ACCCGTTGAACCCCATTCGTGA | | | GCCTCACTAAACCATCCAATCGG |  |  |
| **Primers for amplification of sgRNA-targeted sequence of SPOP/Caprin1 gene** | | | | | |  |  |
| SPOP | | CCTCTCTTGAGTCTGATTTCCA | | | AGAGTTGAACAAAGAGGAGAACAT |  |  |
| Caprin1 | | AGAGGATTGTGAGGGTTTTGTCA | | | TCCTGGTACTTAGAAACGGCATC |  |  |
| **­­­­Sequences of shRNAs** | | | | | |  |  |
| Gene name | | Sequence | | | |  |  |
| shSPOP#1 | | CCGGCAAGGTAGTGAAATTCTCCTACTCGAGT  AGGAGAATTTCACTACCTTGTTTTTT | | | |  |  |
| shSPOP#2 | | CCGGCACAAGGCTATCTTAGCAGCTCTCGAG  AGCTGCTAAGATAGCCTTGTGTTTTTT | | | |  |  |
| **Sequences of siRNAs** | | | | | |  |  |
| Gene name | | Sequence | | | |  |  |
| siRBX1#1 | | GAAGCGCUUUGAAGUGAAA | | |  |  |  |
| siRBX1#2 | | GGGAUAUUGUGGUUGAUAA | | |  |  |  |
| siRBX1#3 | | GGAACCACAUUAUGGAUCU | | |  |  |  |
| siRBX1#4 | | CAUAGAAUGUCAAGCUAAC | | |  |  |  |
| siCUL1#1 | | CAACGAAGAGUUCAGGUUU | | |  |  |  |
| siCUL1#2 | | CGAGGAAGACCGCAAACUA | | |  |  |  |
| siCUL1#3 | | AGACAGUGCUUGAUGUUCA | | | |  |  |
| siCUL1#4 | | CAUAGAAGACAAAGACGUA | | |  |  |  |
| siCUL2#1 | | GGAAGUGCAUGGUAAAUUU | | |  |  |  |
| siCUL2#2 | | CAUCCAAGUUCAUAUACUA | | |  |  |  |
| siCUL2#3 | | GCAGAAAGACACACCACAA | | |  |  |  |
| siCUL2#4 | | UGGUUUACCUCAUAUGAUU | | |  |  |  |
| siCUL3#1 | | GAGAAGATGTACTAAATTC | | |  |  |  |
| siCUL3#2 | | CGACAGAAAACATGAGATA | | |  |  |  |
| siCUL3#3 | | GAAAGTAGACGACGACAGA | | |  |  |  |
| siCUL3#4 | | GAGATCAAGTTGTACGTTA | | |  |  |  |
| siCUL4A#1 | | GCACAGAUCCUUCCGUUUA | | |  |  |  |
| siCUL4A#2 | | GAACAGCGAUCGUAAUCAA | | |  |  |  |
| siCUL4A#3 | | GCAUGUGGAUUCAAAGUUA | | |  |  |  |
| siCUL4A#4 | | GCGAGUACAUCAAGACUUU | | |  |  |  |
| siCUL4B#1 | | UAAAUAACCUCCUUGAUGA | | | |  |  |
| siCUL4B#2 | | CAGAAGUCAUUAAUUGCUA | | | |  |  |
| siCUL4B#3 | | CGGAAAGAGUGCAUCUGUA | | |  |  |  |
| siCUL4B#4 | | GCUAUUGGCCGACAUAUGU | | |  |  |  |
| siCUL5#1 | | GACACGACGUCUUAUAUUA | | |  |  |  |
| siCUL5#2 | | GCAAAUAGAGUGGCUAAUA | | |  |  |  |
| siCUL5#3 | | UAAACAAGCUUGCUAGAAU | | |  |  |  |
| siCUL5#4 | | CGUCUAAUCUGUUAAAGAA | | |  |  |  |
| **Sequences of sgRNAs** | | | | | |  |  |
| Gene name | | Sequence | | | |  |  |
| SPOP | | CAAGCTTACCCTCTTCTGCG | | |  |  |  |
| Caprin1 | | AGTGCCAATATTGTCCGAAG | | | |  |  |
| **Antibody&Chemicals** | | | | | |  |  |
| No. | Name | | Species | Cat. No | Source |  |  |
| 1 | Anti-SPOP | | Rabbit | Ab137537 | Abcam |  |  |
| 2 | Anti-SPOP | | Rabbit | 16750-1-AP | PTG |  |  |
| 3 | Anti-Caprin1 | | Rabbit | 15112-1-AP | PTG |  |  |
| 4 | Anti-Caprin1 | | Mouse | 66352-1-Ig | PTG |  |  |
| 5 | Anti-G3BP1 | | Mouse | 611126 | BD Biosciences |  |  |
| 6 | Anti-G3BP1 | | Rabbit | Ab181149 | Abcam |  |  |
| 7 | Anti-G3BP1 | | Rabbit | SC-81940 | Santa Cruz |  |  |
| 8 | Anti-PABP | | Rabbit | Ab21060 | Abcam |  |  |
| 9 | Anti-EIF4G1 | | Rabbit | A7552 | abclonal |  |  |
| 10 | Anti-EIF3B | | Rabbit | A10259 | abclonal |  |  |
| 11 | Anti-BRD4 | | Rabbit | A301-985A100 | BETHYL |  |  |
| 12 | Anti-DEK | | Rabiit | 16448-1-AP | PTG |  |  |
| 13 | Anti-CASP3 | | Rabbit | 9665 | Cell signaling |  |  |
| 14 | Anti-cleaved CASP3 | | Rabbit | 9664 | Cell signaling |  |  |
| 15 | Anti-CASP7 | | Rabbit | 12827 | Cell signaling |  |  |
| 16 | Anti-cleaved CASP7 | | Rabbit | 8438 | Cell signaling |  |  |
| 17 | Anti-PARP1 | | Rabbit | Ab32138 | Abcam |  |  |
| 18 | Anti-puromycin | | Rabbit | Clone 12D10 | Millipore |  |  |
| 19 | Anti-Myc | | Mouse | M192-7 | MBL |  |  |
| 20 | Anti-FLAG,M2 | | Mouse |  | Sigma |  |  |
| 21 | Anti-FLAG | | Mouse | M185-7 | MBL |  |  |
| 22 | Anti-HA | | Mouse | M180-7 | MBL |  |  |
| 23 | Anti-Actin | | Rabbit | AC028 | abclonal |  |  |
| 24 | Anti-GAPDH | | Mouse | 60004-1-Ig | proteintech |  |  |
| 25 | Anti-Histone H3 | | Rabbit | 4499 | Cell signaling |  |  |
| 26 | MG132 | |  | S2619 | Selleckchem |  |  |
| 27 | Bortezomib | |  | S1013 | Selleckchem |  |  |
| 28 | Docetaxel | |  | S1148 | Selleckchem |  |  |
| 29 | puromycin | |  | S7417 | Selleckchem |  |  |
| ­30 | Sodium arsenite | |  | 80115461 | China National Pharmaceutical |  |  |
| 31 | CHX | |  | HY-B0713 | MCE |  |  |

**Additional file 2: Table S2 SPOP mutation status, Caprin1 IHC scores in 131 cases of prostate cancer specimens and the associated clinical information.**

| NO­­­ | Relative expression level of CAPRIN1 | SPOP Status | CAPRIN1  IHC intensity | Age | BMI  (kg/m 2) | PSA level  (ng/ml) | Hyper-  tension | Type 2 DM | Biopsy  Gleason score | Clinical  stage | Pathologic  stage | Prostatectomy  specimen  Gleason  score |
| --- | --- | --- | --- | --- | --- | --- | --- | --- | --- | --- | --- | --- |
| 1 | 21.2759131 | WT/WT | 1 | 65 | 23.88 | 69.89 | 0 | 0 | 9 | T1 | pT2 | 7 |
| 2 | 7.12121042 | F102C/WT | 3 | 78 | 26.81 | 22.75 | 0 | 0 | 7 | T2 | pT3 | 7 |
| 3 | 2.85110203 | WT/WT | 1 | 65 | 23.2 | 14.14 | 0 | 0 | 7 | T2 | pT2 | 7 |
| 4 | 2.13039811 | WT/WT | 1 | 64 | 25.39 | 52.23 | 1 | 0 | 8 | T2 | pT3 | 9 |
| 5 | 7.49243913 | WT/WT | 2 | 78 | 25.59 | 8.01 | 1 | 0 | 7 | T1 | pT2 | 7 |
| 6 | 1.35566351 | F102Y/WT | 2 | 68 | 24.38 | 9.85 | 0 | 0 | 7 | T2 | pT3 | 10 |
| 7 | 2.22828951 | WT/WT | 1 | 69 | 23.26 | 12.8 | 1 | 0 | 7 | T1 | pT2 | 7 |
| 8 | 11.6800421 | F125C/WT | 2 | 68 | 26.5 | 9.68 | 0 | 1 | 7 | T2 | pT4 | 7 |
| 9 | 2.30074971 | WT/WT | 1 | 73 | 23.23 | 23.62 | 1 | 0 | 7 | T2 | pT3 | 7 |
| 10 | 9.77437702 | WT/WT | 1 | 75 | 26.33 | 18.47 | 0 | 0 | 7 | T2 | pT2 | 7 |
| 11 | 8.50505212 | WT/WT | 0 | 70 | 28.37 | 8.68 | 1 | 0 | 6 | T2 | pT2 | 7 |
| 12 | 14.1808153 | WT/WT | 2 | 74 | 25.26 | 63.83 | 1 | 0 | 7 | T2 | pT3 | 9 |
| 13 | 96.2097507 | WT/WT | 1 | 76 | 23.44 | 11.56 | 1 | 0 | 7 | T1 | pT2 | 6 |
| 14 | 9.73739581 | WT/WT | 1 | 55 | 20.76 | 87.11 | 0 | 0 | 7 | T2 | pT4 | 7 |
| 15 | 4.93230317 | F133L/WT | 3 | 61 | 25.1 | 7.9 | 0 | 0 | 7 | T2 | pT2 | 9 |
| 16 | 21.4791824 | WT/WT | 1 | 65 | 24.91 | 9.03 | 1 | 0 | 8 | T1 | pT3 | 7 |
| 17 | 5.59224136 | WT/WT | 2 | 67 | 25.8 | 39.6 | 0 | 0 | 7 | T1 | pT3 | 7 |
| 18 | 5.5864195 | WT/WT | 2 | 62 | 20.07 | 7.96 | 0 | 0 | 7 | T1 | pT2 | 7 |
| 19 | 20.5955102 | WT/WT | 1 | 78 | 27.68 | 35.43 | 0 | 0 | 9 | T1 | pT2 | 8 |
| 20 | 131.829009 | WT/WT | 1 | 56 | 25.31 | 5.96 | 0 | 0 | 9 | T2 | pT3 | 9 |
| 21 | 1 | WT/WT | 0 | 68 | 22.58 | 7.49 | 1 | 0 | 6 | T2 | pT2 | 6 |
| 22 | 5.27764299 | WT/WT | 1 | 71 | 24.91 | 8.76 | 1 | 0 | 6 | T2 | pT2 | 6 |
| 23 | 4.74084194 | W131G/WT | 2 | 70 | 23.44 | 31.19 | 0 | 0 | 8 | T2 | pT2 | 6 |
| 24 | 54.5681866 | F102C/WT | 3 | 60 | 22.86 | 9.94 | 1 | 0 | 9 | T1 | pT3 | 7 |
| 25 | 16.5441313 | WT/WT | 1 | 54 | 20.62 | 13 | 0 | 0 | 7 | T1 | pT2 | 9 |
| 26 | 5.90836628 | F133V/WT | 2 | 73 | 23.15 | 21.79 | 0 | 1 | 9 | T1 | pT2 | 7 |
| 27 | 3.07802055 | F133L/WT | 3 | 66 | 25.82 | 70.97 | 0 | 0 | 8 | T2 | pT3 | 9 |
| 28 | 4.94047241 | W131C/WT | 3 | 67 | 24.093 | 39.83 | 0 | 0 | 8 | T2 | pT2 | 9 |
| 29 | 11.0984446 | WT/WT | 1 | 77 | 20.52 | 13.5 | 1 | 0 | 7 | T2 | pT2 | 7 |
| 30 | 15.4765923 | F133L/WT | 3 | 76 | 27.18 | 9.87 | 1 | 0 | 8 | T1 | pT2 | 9 |
| 31 | 17.7891371 | WT/WT | 1 | 61 | 23.88 | 14.87 | 0 | 0 | 7 | T2 | pT3 | 8 |
| 32 | 7.98540949 | WT/WT | 1 | 60 | 25.61 | 10.67 | 0 | 0 | 6 | T1 | pT2 | 7 |
| 33 | 4.56850886 | W131G/WT | 1 | 51 | 22.95 | 27.04 | 0 | 0 | 8 | T2 | pT3 | 7 |
| 34 | 13.3312117 | WT/WT | 1 | 71 | 22.32 | 21.37 | 0 | 1 | 7 | T2 | pT2 | 7 |
| 35 | 5.45568764 | WT/WT | 1 | 77 | 25.39 | 47.43 | 1 | 1 | 7 | T2 | pT3 | 7 |
| 36 | 11.3162376 | WT/WT | 1 | 74 | 24.49 | 26.9 | 0 | 1 | 6 | T2 | pT4 | 7 |
| 37 | 79.6746374 | WT/WT | 2 | 75 | 23.88 | 12.57 | 1 | 0 | 7 | T2 | pT2 | 7 |
| 38 | 7.81983262 | WT/WT | 3 | 69 | 24.61 | 89.63 | 1 | 0 | 7 | T2 | pT2 | 9 |
| 39 | 186.890752 | WT/WT | 2 | 62 | 29.41 | 7.95 | 1 | 1 | 6 | T2 | pT2 | 7 |
| 40 | 280.39913 | WT/WT | 1 | 50 | 25.39 | 8.01 | 0 | 0 | 6 | T2 | pT2 | 6 |
| 41 | 22.201254 | WT/WT | 1 | 66 | 25.51 | 6.78 | 0 | 0 | 7 | T2 | pT2 | 7 |
| 42 | 1399.81278 | WT/WT | 2 | 60 | 28.34 | 10.09 | 0 | 0 | 7 | T1 | pT2 | 6 |
| 43 | 282.1875 | WT/WT | 1 | 70 | 22.0386 | 90.81 | 0 | 0 | 8 | T2 | pT3 | 9 |
| 44 | 1675.56287 | WT/WT | 1 | 72 | 21.4533 | 25.81 | 1 | 1 | 6 | T1 | pT2 | 6 |
| 45 | 432.911413 | WT/WT | 1 | 71 | 21.77 | 51.65 | 0 | 0 | 9 | T1 | pT2 | 9 |
| 46 | 101.541489 | WT/WT | 3 | 62 | 21.11 | 5.8 | 0 | 0 | 6 | T2 | pT2 | 7 |
| 47 | 910.1477 | WT/WT | 1 | 72 | 21.11 | 7.4 | 0 | 0 | 7 | T2 | pT3 | 7 |
| 48 | 43.4009577 | WT/WT | 1 | 74 | 22.84 | 18.74 | 1 | 0 | 7 | T2 | pT2 | 7 |
| 49 | 70.999814 | WT/WT | 1 | 55 | 26.3 | 7.44 | 0 | 0 | 7 | T1 | pT2 | 7 |
| 50 | 810.848124 | WT/WT | 1 | 65 | 26.22 | 7.6 | 0 | 0 | 8 | T2 | pT3 | 9 |
| 51 | 98.7698055 | WT/WT | 1 | 80 | 21.34 | 16.54 | 1 | 0 | 9 | T2 | pT2 | 8 |
| 52 | 130.706499 | WT/WT | 1 | 61 | 25.35 | 14.58 | 0 | 0 | 7 | T2 | pT2 | 8 |
| 53 | 112.951959 | WT/WT | 1 | 50 | 27.1 | 14.87 | 0 | 0 | 7 | T1 | pT2 | 7 |
| 54 | 37.1798091 | WT/WT | 3 | 55 | 25.76 | 30.82 | 1 | 0 | 6 | T1 | pT2 | 6 |
| 55 | 63.414333 | WT/WT | 1 | 73 | 19.61 | 13.11 | 0 | 0 | 9 | T1 | pT2 | 7 |
| 56 | 70.1725098 | WT/WT | 0 | 68 | 24.69 | 14 | 0 | 0 | 9 | T2 | pT4 | 9 |
| 57 | 21.3150993 | F133V/WT | 2 | 67 | 26.3 | 13.95 | 0 | 0 | 7 | T1 | pT2 | 7 |
| 58 | 88.5787166 | WT/WT | 0 | 69 | 23.88 | 2.52 | 0 | 0 | 7 | T2 | pT2 | 7 |
| 59 | 42.6464165 | WT/WT | 1 | 72 | 24.22 | 29.96 | 0 | 0 | 7 | T1 | pT2 | 7 |
| 60 | 41.6849232 | WT/WT | 0 | 69 | 22.23 | 4.5 | 0 | 0 | 7 | T2 | pT2 | 7 |
| 61 | 62.5573986 | WT/WT | 1 | 72 | 25.47 | 21.91 | 0 | 0 | 7 | T2 | pT2 | 6 |
| 62 | 29.0348783 | WT/WT | 1 | 56 | 22.86 | 4 | 1 | 0 | 7 | T1 | pT2 | 7 |
| 63 | 972.087121 | F102S/WT | 2 | 72 | 23.44 | 48.13 | 1 | 1 | 8 | T1 | pT4 | 8 |
| 64 | 1516.43337 | F102S/WT | 3 | 79 | 23.44 | 21.2 | 0 | 0 | 9 | T2 | pT3 | 7 |
| 65 | 2103.68385 | WT/WT | 1 | 64 | 24.8 | 10.3 | 1 | 1 | 6 | T2 | pT2 | 7 |
| 66 | 361.960664 | WT/WT | 2 | 74 | 25.86 | 39.5 | 1 | 0 | 7 | T2 | pT2 | 7 |
| 67 | 1582.49034 | WT/WT | 1 | 67 | 23.03 | 46.2 | 0 | 0 | 8 | T1 | pT3 | 8 |
| 68 | 9816.52455 | WT/WT | 3 | 72 | 27.47 | 19.76 | 1 | 0 | 9 | T1 | pT3 | 10 |
| 69 | 167.671976 | WT/WT | 1 | 68 | 31.25 | 47.28 | 1 | 0 | 8 | T2 | pT3 | 9 |
| 70 | 379.881324 | WT/WT | 1 | 63 | 23.53 | 16.84 | 0 | 0 | 8 | T1 | pT3 | 8 |
| 71 | 54.0101036 | WT/WT | 1 | 67 | 27.78 | 7.66 | 0 | 1 | 10 | T1 | pT3 | 9 |
| 72 | 51.4397369 | WT/WT | 1 | 77 | 29.27 | 17.58 | 0 | 0 | 6 | T1 | pT2 | 9 |
| 73 | 222.016297 | WT/WT | 0 | 58 | 24.22 | 24.08 | 0 | 0 | 7 | T2 | pT4 | 9 |
| 74 | 333.03857 | WT/WT | 1 | 77 | 21.48 | 4.62 | 0 | 0 | 8 | T2 | pT2 | 8 |
| 75 | 130.760148 | WT/WT | 1 | 70 | 24.69 | 9.55 | 0 | 0 | 7 | T2 | pT3 | 7 |
| 76 | 275.643889 | WT/WT | 0 | 76 | 20.08 | 9.98 | 1 | 1 | 6 | T2 | pT2 | 7 |
| 77 | 196.841609 | WT/WT | 1 | 70 | 31.77 | 11.56 | 1 | 0 | 9 | T2 | pT2 | 9 |
| 78 | 209.919835 | WT/WT | 2 | 70 | 31.89 | 5.68 | 1 | 0 | 7 | T2 | pT3 | 9 |
| 79 | 197.159697 | WT/WT | 1 | 69 | 24.21 | 6.96 | 1 | 0 | 7 | T2 | pT2 | 7 |
| 80 | 1231.91337 | WT/WT | 1 | 70 | 23.14 | 14.2 | 1 | 0 | 9 | T3 | pT2 | 9 |
| 81 | 4374.71573 | WT/WT | 1 | 69 | 25.71 | 19.7 | 1 | 0 | 7 | T2 | pT2 | 7 |
| 82 | 844.881944 | WT/WT | 0 | 59 | 23.59 | 5.26 | 0 | 0 | 8 | T2 | pT2 | 8 |
| 83 | 857.235594 | WT/WT | 1 | 60 | 25.39 | 20.45 | 1 | 0 | 8 | T2 | pT2 | 9 |
| 84 | 775.614371 | WT/WT | 1 | 68 | 21.48 | 14.76 | 0 | 0 | 7 | T2 | pT2 | 7 |
| 85 | 1565.49252 | WT/WT | 2 | 63 | 26.99 | 17.93 | 0 | 0 | 8 | T2 | pT2 | 8 |
| 86 | 204.809779 | WT/WT | 1 | 75 | 23.66 | 1.29 | 1 | 0 | 9 | T4 | pT3 | 9 |
| 87 | 2129.39798 | WT/WT | 2 | 68 | 23.44 | 18.72 | 0 | 1 | 8 | T2 | pT2 | 7 |
| 88 | 1986.70574 | WT/WT | 1 | 63 | 23.44 | 8.61 | 0 | 0 | 7 | T2 | pT2 | 7 |
| 89 | 10261.3616 | WT/WT | 1 | 69 | 21.71 | 25.72 | 1 | 0 | 8 | T2 | pT3 | 8 |
| 90 | 340.853402 | WT/WT | 0 | 46 | 24.16 | 5.96 | 0 | 0 | 6 | T2 | pT3 | 7 |
| 91 | 12.7604931 | WT/WT | 2 | 60 | 24.91 | 164.9 | 1 | 0 | 9 | T4 | pT4 | 9 |
| 92 | 258.497991 | WT/WT | 1 | 64 | 25.4 | 17.94 | 1 | 0 | 9 | T2 | pT2 | 8 |
| 93 | 86.2285578 | WT/WT | 1 | 74 | 20.94 | 8.38 | 0 | 0 | 7 | T1 | pT2 | 7 |
| 94 | 279.838285 | WT/WT | 2 | 69 | 26.2346 | 7.89 | 1 | 0 | 7 | T2 | pT2 | 8 |
| 95 | 10.9431671 | WT/WT | 3 | 77 | 21.1073 | 8.32 | 0 | 0 | 9 | T2 | pT3 | 8 |
| 96 | 93.3666572 | WT/WT | 1 | 63 | 20.5191 | 21.674 | 0 | 0 | 6 | T2 | pT3 | 7 |
| 97 | 188.312323 | F102L/WT | 3 | 70 | 24.3375 | 15.312 | 0 | 0 | 8 | T2 | pT3 | 8 |
| 98 | 6685.94254 | WT/WT | 1 | 64 | 20.6193 | 15.8 | 0 | 0 | 6 | T2 | pT3 | 6 |
| 99 | 585.217487 | WT/WT | 1 | 69 | 17.301 | 7.64 | 1 | 0 | 8 | T4 | pT3 | 9 |
| 100 | 287.168275 | WT/WT | 2 | 64 | 33.2031 | 19.673 | 0 | 0 | 8 | T2 | pT2 | 8 |
| 101 | 119.17355 | WT/WT | 2 | 68 | 26.1224 | 44.457 | 0 | 0 | 8 | T1 | pT3 | 7 |
| 102 | 50.975418 | K134N/WT | 3 | 58 | 25.0593 | 16.833 | 1 | 1 | 7 | T2 | pT3 | 7 |
| 103 | 2029.26568 | WT/WT | 1 | 68 | 24.0741 | 78.128 | 0 | 0 | 7 | T3 | pT3 | 9 |
| 104 | 150.555354 | WT/WT | 2 | 74 | 20.8209 | 17.361 | 0 | 0 | 6 | T1 | pT2 | 7 |
| 105 | 401.773361 | WT/WT | 2 | 65 | 27.6817 | 23.39 | 1 | 0 | 7 | T1 | pT2 | 7 |
| 106 | 1435.61617 | WT/WT | 1 | 66 | 21.4844 | 55.98 | 0 | 1 | 9 | T1 | pT2 | 9 |
| 107 | 989.215596 | FI 33V/WT | 2 | 64 | 23.4509 | 9.587 | 0 | 0 | 7 | T2 | pT3 | 7 |
| 108 | 382.6276 | F102I/WT | 3 | 74 | 27.3356 | 14.536 | 1 | 1 | 8 | T2 | pT3 | 7 |
| 109 | 1188.92229 | WT/WT | 2 | 78 | 25.9516 | 23.494 | 1 | 0 | 6 | T3 | pT3 | 7 |
| 110 | 1907.67025 | WT/WT | 2 | 74 | 23.5294 | 15.64 | 0 | 0 | 7 | T2 | pT2 | 7 |
| 111 | 145.65096 | WT/WT | 1 | 72 | 24.6755 | 13.35 | 1 | 0 | 9 | T2 | pT3 | 9 |
| 112 | 617.521766 | WT/WT | 1 | 75 | 25.0593 | 7.6 | 0 | 0 | 6 | T2 | pT3 | 7 |
| 113 | 2697.13562 | WT/WT | 2 | 80 | 20.4783 | 11.428 | 0 | 0 | 7 | T1 | pT3 | 7 |
| 114 | 1736.5332 | WT/WT | 1 | 64 | 24.0569 | 5.473 | 0 | 0 | 6 | T1 | pT2 | 7 |
| 115 | 812.9258 | WT/WT | 3 | 78 | 19.0311 | 19.104 | 0 | 0 | 7 | T2 | pT2 | 7 |
| 116 | 51.0324784 | WT/WT | 2 | 69 | 23.8754 | 95.3 | 1 | 0 | 7 | T2 | pT3 | 9 |
| 117 | 98.1290522 | WT/WT | 2 | 70 | 21.875 | 37.19 | 0 | 0 | 9 | T3 | pT3 | 9 |
| 118 | 886.080005 | WT/WT | 1 | 65 | 26.2976 | 8.7 | 0 | 0 | 8 | T2 | pT3 | 7 |
| 119 | 3770.0847 | WT/WT | 2 | 60 | 26.5731 | 19.312 | 0 | 0 | 8 | T1 | pT2 | 8 |
| 120 | 298.783185 | WT/WT | 1 | 60 | 22.3094 | 1161.92 | 1 | 1 | 10 | T2 | pT3 | 8 |
| 121 | 4384.3403 | FI 33V/WT | 3 | 79 | 24.2215 | 100 | 1 | 0 | 9 | T2 | pT3 | 9 |
| 122 | 813.96716 | WT/WT | 2 | 68 | 22.1297 | 9.11 | 1 | 0 | 6 | T1 | pT3 | 7 |
| 123 | 954.473239 | WT/WT | 3 | 76 | 28.0843 | 9.44 | 1 | 0 | 9 | T1 | pT2 | 8 |
| 124 | 898.62555 | WT/WT | 1 | 72 | 19.052 | 17.007 | 0 | 0 | 6 | T1 | pT2 | 7 |
| 125 | 244.798919 | WT/WT | 1 | 57 | 21.2245 | 22 | 1 | 1 | 6 | T2 | pT2 | 8 |
| 126 | 445.244478 | WT/WT | 2 | 63 | 25.3906 | 8.062 | 0 | 0 | 9 | T1 | pT2 | 9 |
| 127 | 1366.57443 | WT/WT | 1 | 66 | 24.2215 | 30.56 | 0 | 0 | 7 | T2 | pT3 | 7 |
| 128 | 402.332616 | WT/WT | 1 | 70 | 22.0386 | 21.78 | 0 | 0 | 8 | T3 | pT2 | 7 |
| 129 | 102.0158 | WT/WT | 0 | 59 | 23.3844 | 22.88 | 0 | 0 | 9 | T1 | pT3 | 9 |
| 130 | 81.0601269 | WT/WT | 1 | 70 | 25.3515 | 14 | 1 | 0 | 8 | T2 | pT2 | 7 |
| 131 | 104.83864 | WT/WT | 1 | 76 | 28.6501 | 29.74 | 1 | 1 | 6 | T1 | pT2 | 9 |

**Supplementary Table 3. Primers , sequences of shRNAs and siRNAs, antibody and chemicals.**

|  | | **PSA** | **Gleason score** | **Pathological T stage** |
| --- | --- | --- | --- | --- |
| Caprin1 IHC intensity | r | 0.173* | 0.070 | 0.073 |
|  | P value | 0.049 | 0.425 | 0.409 |
|  | N | 131 | 131 | 131 |

***r*= spearman correlation coefficient; ^*^ *P*<0.05.**
